# The effect of COVID-19 and sex differences on natural killer cell cytotoxicity

**DOI:** 10.3389/fcimb.2025.1635043

**Published:** 2025-09-22

**Authors:** Arushi Dagar, Maria J. Polyak, Adley C. H. Mok, David Feehan, Michael Potemkin, Alain Tremblay, Christopher H. Mody

**Affiliations:** ^1^ Department of Microbiology, Immunology, and Infectious Diseases, Cumming School of Medicine, Calgary, AB, Canada; ^2^ Calvin, Phoebe, and Joan Snyder Institute for Chronic Diseases, University of Calgary, Calgary, AB, Canada; ^3^ Department of Medicine, University of Calgary, Calgary, AB, Canada

**Keywords:** COVID-19, cytotoxic effector molecules, granule trafficking, natural killer (Nk) cell, cellular cytotoxicity, immunology, sex differences, SARS-CoV-2

## Abstract

COVID-19 has caused more than 7 million deaths worldwide, and according to the World Health Organization, it continues to result in more than 1000 reported deaths per month at the time of this writing. It is crucial to understand the immune response to COVID-19 since the virus continues to persist. Natural killer (NK) cells play a critical role in the immune defense against viral infections, including COVID-19. While it is well documented that infected patients have a reduction in lymphocytes and NK cells, gaps in knowledge exist regarding the function of NK cells. To study the function of NK cells in patients hospitalized with COVID-19, peripheral blood was obtained from patients admitted to the medical (non-ICU) wards at a large tertiary hospital. We demonstrated a decrease in the mature cytotoxic subset of NK cells within the peripheral blood of patients hospitalized with COVID-19. We also observed a notable reduction in the cytotoxic function of NK cells against tumor targets. We examined the mechanisms leading to NK cell killing. We found reductions in the intracellular levels of effector molecules, the degranulation of cytotoxic granules, and the extracellular concentrations of released effector molecules. We identified dysfunctional intracellular granule trafficking required to position the granules for degranulation, which would be consistent with the reduced release of effector molecules. We found clusters of inhibitory receptors were upregulated in subsets of NK cells, in keeping with inhibition of cytotoxicity. Additionally, males with COVID-19 showed NK cell defects compared to healthy males, while no significant differences were observed in females. Our findings highlight defects in cytolytic effector molecules, granule trafficking and release, and increased expression of inhibitory receptors on NK cells in patients hospitalized with COVID-19, in addition to a sex difference in cytolytic function, which contributes to defective NK cell function in COVID-19.

## Introduction

### Importance of COVID-19

The COVID-19 pandemic began in December 2019 with the emergence of a novel member of the coronavirus family, SARS-CoV-2. As of July 14, 2024, more than 778 million people have been infected globally, resulting in more than seven million deaths worldwide (World Health Organization, 2024). The infection spans the spectrum from asymptomatic to severe respiratory disease, leading to hospitalization, admission to the ICU and death (Ziehr et al., 2020; Qi et al., 2021; Nanwani-Nanwani et al., 2022). The devastating impact of COVID-19 has led to intense interest in the host’s defense against the infection, involving organ-specific defense and innate and adaptive immunity, including natural killer (NK) cells.

### The role of NK cells in host defense and control of activation

Natural killer (NK) cells are innate lymphoid cells that serve as one of the first lines of defense against tumor cells and microbial pathogens, including bacteria, fungi, parasites, and viruses (Abel et al., 2018; Garcia-Peñarrubia et al., 1989; Kiessling et al., 1975; Lodoen and Lanier, 2006; Schmidt et al., 2013). Unlike other cytotoxic lymphocytes, natural killer cells do not require previous exposure to recognize and deploy their cytotoxic products to attack and kill pathogens, pathogen-infected or malignantly transformed cells (Kiessling et al., 1975). Viruses and tumors can cause a reduction in inhibitory MHC-I molecules on the target cells, making them susceptible to NK cell targeting through activating receptors (See et al., 1997).

### Effect of viruses on NK cell activity

Some viruses stimulate NK cell activity, while others suppress their function, leading to contrasting effects on host defense. For instance, the human immunodeficiency virus and respiratory syncytial virus have been shown to enhance NK cell activity (Richard et al., 2010; van Erp et al., 2019). By contrast, other viruses, such as influenza A, human pegivirus, and vaccinia, reduce NK cell function in infected individuals (Kirwan et al., 2006; Mao et al., 2010; Chivero et al., 2015). Different viruses can manipulate various pathways of activation and inhibition in NK cells. For example, human cytomegalovirus and human T lymphotropic virus type 1 enhance NK cell function through augmented degranulation and increased IFN-γ production. However, they can also inhibit NK cell function by altering receptor expression, including checkpoint proteins (Béziat et al., 2013; Tschan-Plessl et al., 2016; Amorim et al., 2019). Consequently, it is necessary to understand the sequence of events during NK cell function required to predict the effect of viruses under different conditions.

### Pathways leading to cytotoxicity

When an NK cell encounters a tumor or virus-infected cell, it kills the target cell by releasing cytotoxic effector molecules. These molecules, such as perforin, granulysin, and granzymes, are released from the NK cells’ granules, also called vesicles or secretory lysosomes. This degranulation event requires the receptor activation signal to be stronger than the inhibitory signal. This triggers cytoskeletal rearrangement, which is necessary for the proper trafficking of vesicles within the NK cells toward the immunological synapse, where they can release their cytotoxic proteins.

Perforin damages or creates pores in the target cell membrane, allowing the entry of granzymes (Lieberman, 2003; Trapani and Bird, 2008). Additionally, granulysin associates with the plasma membrane of tumor cells, causing mitochondrial damage and calcium overload, leading to cell death (Okada et al., 2003). Granzymes, including Granzyme A, B and K, are a family of enzymes that induce caspase-dependent cell death (Beresford et al., 2001; Chowdhury and Lieberman, 2008; Martinvalet et al., 2005; Zhao et al., 2007). While studies have described the intracellular levels of granzymes A, B and perforin in patients with COVID-19, it still needs to be clarified whether the extracellular concentration of these molecules is altered upon stimulation.

### NK cell activity in COVID-19

NK cells are dysfunctional in severe COVID-19. In hospitalized patients with severe COVID-19 (grade 6 to 9 on the WHO progression scale), the number of NK cells is reduced, and the expression of IFN-stimulated genes and genes involved in IFN-α signaling is increased, leading to defective NK cell cytotoxicity (Marshall et al., 2020; Zheng et al., 2020; Krämer et al., 2021). IFN-α stimulates NK cell function and can downregulate IFN-γ production (Krämer et al., 2021; Lee et al., 2019). When considering intracellular effector molecule content, there is less consensus about whether granzyme B and perforin expression increase or decrease (Maucourant et al., 2020; Zheng et al., 2020; Witkowski et al., 2021). There is also an impairment in cytotoxic degranulation assessed by CD107a presentation (Krämer et al., 2021; Witkowski et al., 2021).

Independent studies have looked at limited panels of activating and inhibitory receptors, and the conclusions drawn so far show an exhausted phenotype based on the expression of NKG2D, NKG2A, PD-1, CTLA-4, and TIM-3 (Zheng et al., 2020; Varchetta et al., 2021; Ustiuzhanina et al., 2023). Transcriptional analysis has proposed that these defects are due to a transforming growth factor-β (TGF-β) signature leading to reduced cell-cell adhesion, granule exocytosis and cytotoxicity (Witkowski et al., 2021). However, a systematic assessment of the sequence of intracellular events leading to NK cell cytotoxicity and the expression of inhibitory receptors as possible mechanisms of defective cytotoxicity in patients hospitalized with COVID-19 (PHWC-19) has not been performed.

### Demographic and clinical factors affecting NK cell responses in COVID-19

Demographic and clinical factors can affect NK cell intracellular mechanisms. Sex differences are present across many aspects of the immune system and are essential to understand and treat infections and immunologic disorders fully. Males are more likely to become hospitalized when infected with COVID-19 and comprise the majority of all age and ethnic groups in hospitalized patients (Qi et al., 2021). There are differences in receptor and cytokine expression, which could be the basis for sex differences in the immune response to SARS-CoV-2. Despite the relationship between sex differences and the severity of COVID-19, there is a lack of information about NK cell function in males and females hospitalized with COVID-19.

The ancestral strain of SARS-CoV-2 predates the subsequent variants of concern (VOCs) that evolved later in the pandemic. These variants, including Alpha, Beta, Gamma, Delta, and Omicron, are progressively more transmissible due to genetic adaptation and external pressures such as vaccine-induced and natural immunity (Carabelli et al., 2023; Jacobs et al., 2023). It has been shown that the CD56^dim^ CD16^bright^ NK cells from patients infected with Omicron BA.2 demonstrated greater IFN-γ secretion and cytotoxicity in previously vaccinated individuals compared to unvaccinated controls (Peng et al., 2023). However, there was no comparison of NK cell functioning in patients infected with earlier VOCs.

Dexamethasone, a corticosteroid, was introduced as an anti-inflammatory medication to reduce the progression of organ injury and failure that could result in death (The Recovery Collaborative Group, 2021). Moderate administration of dexamethasone benefits PHWC-19 with respiratory support (supplemental oxygen or ventilation), and no advantages have been found in patients without respiratory aids (The Recovery Collaborative Group, 2021). There have been studies regarding the effects of corticosteroids, with some studies showing an enhancement of proliferation and cytokine production of NK cells while others showing reduced cytotoxicity (Piccolella et al., 1986; Morgan and Davis, 2017; Chen et al., 2018). The role of dexamethasone as an independent variable on NK cell function during COVID-19 infection has not been investigated. One study found no difference in the cytotoxicity of NK cells from patients before and after the establishment of dexamethasone as a standard treatment for COVID-19, but did not investigate further (Witkowski et al., 2021).

### The significance and approach

We utilized antibody labelling and mass cytometry to determine the percentages of different blood NK cell subsets and other lymphocytes reduced in PHWC-19. NK cells from PHWC-19 were co-cultured with a tumor cell target to compare cytotoxicity with NK cells from healthy subjects. This led to our investigation of the mechanisms involved in NK cell cytotoxicity. Using flow and mass cytometry, we found that NK cells from PHWC-19 had reduced effector molecule expression, degranulation, and release using flow cytometry and ELISA. We used live cell imaging to establish that granule trafficking and polarization were defective in NK cells from PHWC-19, which is critical for granule release and effectiveness. We used a panel of antibodies of activating and inhibitory receptors to show that inhibitory receptor expression was enhanced, which could affect downstream granule trafficking, granule content, and degranulation. Lastly, we explored the role of demographic and clinical factors like age, sex, VOCs, prior vaccination and dexamethasone treatment on NK cell functioning to understand how NK cells are affected by COVID-19.

## Materials and methods

### Study participants

Peripheral blood samples were collected from forty-four patients hospitalized in a medical ward at Foothills Medical Centre, Calgary, Canada, after informed consent was obtained between November 2020 and September 2023. The patients were categorized within the moderate range of 4 or 5 on the WHO clinical progression scale for COVID-19 disease severity (Marshall et al., 2020). These patients presented with clinical features requiring hospitalization but were not admitted to the intensive care unit. Patients were excluded if they had major comorbidities that might affect study outcomes, were immunosuppressed, including having an active malignancy or receiving immunosuppressive drugs. Characteristics such as age, sex, vaccination status, dexamethasone treatment, and variants at the time of hospitalization are summarized in **Table 1**. When patient samples were obtained, peripheral blood samples were collected on the same day from forty-five healthy donors at the Cumming School of Medicine to act as the paired negative controls.

**Table 1:** Characteristics of study subjects.

Characteristic^1^	Non-ICU hospitalized patients (N = 44)	Healthy controls (N = 45)
Age group – No.		
Mean ± SD	63 ± 19	36 ± 19
20-40:	6	32
41-60:	13	5
61-80+:	25	8
Male Sex – No. (%)	24 (54%)	21 (47%)
Dexamethasone Treatment – No.		
Yes:	22	N/A
No:	22	N/A
Vaccination Status – No.		
Yes:	19	34
No:	21	7
Unknown:	4	4
Presumed and Confirmed Variants – No.		
Ancestral:	9	N/A
Alpha:	7	N/A
Delta:	15	N/A
Omicron:	13	N/A

Participant demographics in the healthy and non-ICU hospitalized COVID-19 cohort. Information includes age, sex, dexamethasone treatment, vaccination status, and confirmed and presumed variant status based on the timing of diseases.

### Primary natural killer cell isolation from whole blood

Peripheral blood mononuclear cells (PBMCs) were isolated from whole blood using the Ficoll-Paque^®^ separation technique (Millipore Sigma, Germany). PBMCs were used for flow cytometry studies, while primary NK cells (pNK) were isolated by negative selection using magnetic beads (Miltenyi Biotec, Germany). Three mL of whole blood was put aside for mass cytometry.

### NK cellular cytotoxicity

NK cell cytotoxicity was assessed using 721.221 tumor cells (a gift from Dr. Nicole Bernard, McGill University, Montreal). The 721.221 cells were cultured in RPMI 1640 (Gibco, Thermo Fisher Scientific Inc, Waltham, MA) with 10% fetal bovine serum (FBS) and 1% Penicillin-Streptomycin-Glutamine (PSQ). The 721.221 cells were stained with the carboxyfluorescein succinimidyl ester (CFSE) (Millipore Sigma, Germany) as a cell tracking dye to distinguish them from the primary cells. The cells were co-cultured with NK cells in RPMI 1640 with 10% FBS, 1% non-essential amino acids, 1% Na pyruvate, 1% PSQ at 5:1 and 10:1 effector-to-target (E:T) ratios for 4 hours before adding 7-amino actinomycin D (7AAD) (Invitrogen, Thermo Fisher Scientific Inc, Waltham) and analyzed by flow cytometry (Guava EasyCyte, EMD Millipore, Danvers, MA). The positive control for cell death was obtained by fixing 721.221 cells with 4% paraformaldehyde (PFA) followed by Triton-X100 (0.1% in PBS). The percentage of cell death is analyzed using the FlowJo LLC program by examining the percentage of cells that are double positive for the CFSE and 7AAD stains.

### Antibody labelling and flow cytometry for cell surface markers, intracellular cytotoxic effector molecules, and checkpoint inhibitor receptors

PBMCs were fixed with 4% PFA and permeabilized with saponin (0.1%). Intracellular effector proteins granulysin, perforin, and granzymes A, B, and K, and checkpoint proteins LAG-3, PD-1, TIGIT, and CD94 were labelled with fluorescent antibodies or their respective isotype control antibodies for 30 minutes along with anti-CD56 and anti-CD3 to define NK cells (**Supplementary Table 1**). Geometric mean fluorescence intensity was assessed by flow cytometry (LSRII, BD Biosciences, New Jersey). The healthy and COVID-19 samples were collected and processed simultaneously to limit interexperimental variability. Initially, PBMCs were gated on CD56+ CD3- cell population for NK cells. Histograms were then utilized to determine the percentage of NK cells positive for each marker. Flow cytometry data was analyzed using the FlowJo LLC program.

### NK cell degranulation

NK cell degranulation was assessed as previously described (Islam et al., 2013). The Anti-CD107a fluorescent antibody and the intracellular protein transport inhibitor cocktail Brefeldin A/Monensin were added to the NK cell plus 721.221 cell co-culture for four hours. Cells were fixed with 4% PFA, labelled with CD56 and CD3, and analyzed by flow cytometry (Guava EasyCyte, EMD Millipore, Danvers, MA). These experiments were conducted concurrently in the healthy and COVID-19 samples to pair the results.

### Effector molecule release ELISA assay and analyses

Supernatants from the co-cultures of NK and tumor cells were collected at 4 hours and stored at -80°C prior to measuring levels of released perforin, granzyme A and granzyme B by ELISA. Effector molecule release was measured using the manufacturer’s reagents and instructions (Abcam, Cambridge).

### Granule trafficking

Live-cell imaging of primary NK cells in the presence of 721.221 target cells was used to assess spatial and temporal changes in conjugate formation and granule trafficking. The NK cells were treated with Cresyl violet to label granules (Millipore Sigma, Germany) and tubulin tracker green to label microtubules (Invitrogen, Thermo Fisher Scientific Inc, Waltham, MA). A Ti Eclipse microscope (Nikon Instruments Inc., Melville, NY) was used with an objective lens with a magnification of 60X, a numerical aperture of 1.2 and oil immersion. Images were analyzed using the ImageJ Fiji program following the protocol described previously to measure granule distances to the microtubule-organizing center (MTOC) and the synapse (Hsu et al., 2017). The synapse is defined as the center of the area of contact between the NK cell and the 721.221 tumor cell. The width of the synapse was determined when the plasma membrane of the NK cell and the tumor cell was deformed by the contact and defined as the linear distance between the points of contact of both cells’ plasma membranes. The granule distance is defined as the mean distance from the granules to the closest point on the immunological synapse. Distances of the MTOC to synapse and granule to synapse were determined over time using the Trackmate plugin in ImageJ (Ershov et al., 2022). Imaging in the healthy donor was performed at a frame rate of 1 image every minute for 60 minutes, and imaging in the PHWC-19 was performed at a frame rate of 1 image every minute for 80 minutes. The time to initial polarization was defined as the time between the start of a sustained contact between the NK cell and the tumor cell and the initial decrease in distance of the granules to the synapse.

### Surface receptors assessed by mass cytometry (CyTOF)

The whole blood was diluted at a ratio of 1:1.4 of blood to the PROT1 stabilizer for ten minutes and stored at -80°C until the samples were prepared for mass cytometry (HELIOS, Standard BioTools Inc, San Francisco). The cells were thawed, and the red blood cells were lysed using the PROT1 lysis buffer and washed with a cell staining media (CSM) consisting of phosphate-buffered saline (PBS) (Gibco, Thermo Fisher Scientific Inc, Waltham, MA) with 1% bovine serum albumin (BSA). The samples were fixed, permeabilized and barcoded with the Cell-ID 20-Plex Pd Barcoding Kit (Standard BioTools Inc, San Francisco) according to the manufacturer’s recommendations. After barcoding, the cells were adjusted to have 5 x 10^5^ cells per sample for 3 x 10^6^ cells per tube. Cells were washed, and samples were combined and stained with a surface staining cocktail of lanthanide-labelled antibodies (**Supplementary Table 2**) for 30 mins at room temperature. Cells were reconstituted in 0.3% Saponin/PBS with iridium and 1.6% paraformaldehyde in the fridge overnight. The next day, the cells were washed with CSM and PBS before being aspirated into a pellet and brought to the HELIOS machine for acquisition. All the data was uploaded to the Cytobank program to generate all gates and run all analysis methods, including viSNE for dimensionality reduction and FlowSom for clustering cells. GraphPad Prism (GraphPad Software Inc, CA) was used to conduct the PCA analysis of the data from mass cytometry. Gating strategies for cell populations have been provided in **Supplementary Figure 1**.

### Statistical analysis

Statistical analysis was performed using GraphPad Prism (GraphPad Software Inc, CA). A paired t-test was used to assess cytotoxicity and degranulation after ensuring normal distribution based on a Shapiro-Wilk test. The remaining analysis was done with Mann-Whitney, Wilcoxon matched-pairs signed rank, or Kruskal-Wallis ANOVA based on paired or unpaired samples and the number of comparisons. P<0.05 was considered statistically significant.

## Results

### PHWC-19 have reduced numbers of peripheral blood CD56+ CD3- NK cells

**Figure 1:**
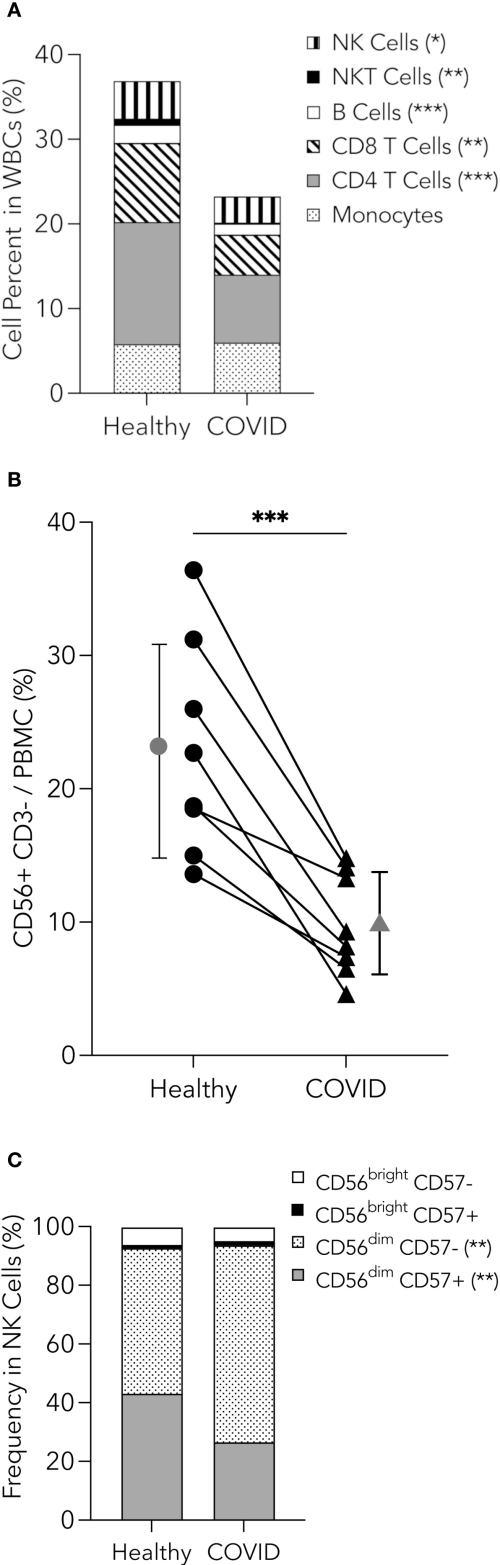
Reduction in peripheral blood mononuclear cells, including NK cells in PHWC-19. **(A)** Pooled relative percentages of immune cell populations (NK cells, NKT cells, B cells, CD8 and CD4 T cells, and monocytes) as a percentage of cells within the CD45+ cells from whole blood collected from healthy donors (N = 15) and PHWC-19 (N = 17), detected by mass cytometry. **(B)** Percentage of CD56+CD3- NK cells within the peripheral blood mononuclear cells (PBMCs). PBMCs were obtained on the same day in PHWC-19 and the controls. Cells were labelled with anti-CD56 and anti-CD3 antibodies and analyzed by flow cytometry to determine abundance. The line connecting the healthy and COVID-19 points represents paired patient and control samples, N = 8. **(C)** Percentages of NK cell subsets based on expressions of CD56 and CD57 within the NK cell population for healthy donors (N = 15) and PHWC-19 (N = 17) detected by single-cell mass cytometry. * = P<0.05, ** = P<0.01, *** = P<0.001; calculated using the Mann-Whitney test.

NK cells play an essential role in the innate immune system during many respiratory diseases. These cells can kill tumors, virus-infected cells, and bacterial and fungal pathogens (Garcia-Peñarrubia et al., 1989; Lodoen and Lanier, 2006; Schmidt et al., 2013). Since the number of NK cells could affect the severity and degree of immune dysfunction in PHWC-19, the peripheral whole blood of PHWC-19 was analyzed using mass cytometry. We found a significant decrease in the percentage of NK cells and, indeed, all mononuclear cells in PHWC-19 compared to healthy donors (**Figure 1A**, gating strategy in **Supplementary Figure 1**). Using flow cytometry, we found the percentage of CD3- CD56+ NK cells in PHWC-19 was lower compared to healthy controls processed on the same day (9.8 ± 3.8% in PHWC-19, 22.8 ± 8.0% in healthy subjects) (**Figure 1B**). We further assessed different NK cell subsets based on the expression of CD56 and CD57. We found that the mature, differentiated, and cytotoxic NK cell subset, CD56^dim^ CD57+, was decreased in PHWC-19, while the immature cytotoxic NK cells, CD56^dim^ CD57- increased (**Figure 1C**) (Fan et al., 2008; Vivier et al., 2008). The less abundant cytokine-producing CD56^bright^ CD57+/- population remained unaltered due to COVID-19 (**Figure 1C**) (Lopez-Vergès et al., 2010; Béziat et al., 2011). Although the reduced numbers and maturity of NK cells may contribute to the observed reduction in cytotoxicity, we were interested in determining whether there is a defect at the cellular level of NK cell function.

### PHWC-19 has decreased cytotoxic abilities in peripheral CD56+ CD3- NK cells

**Figure 2:**
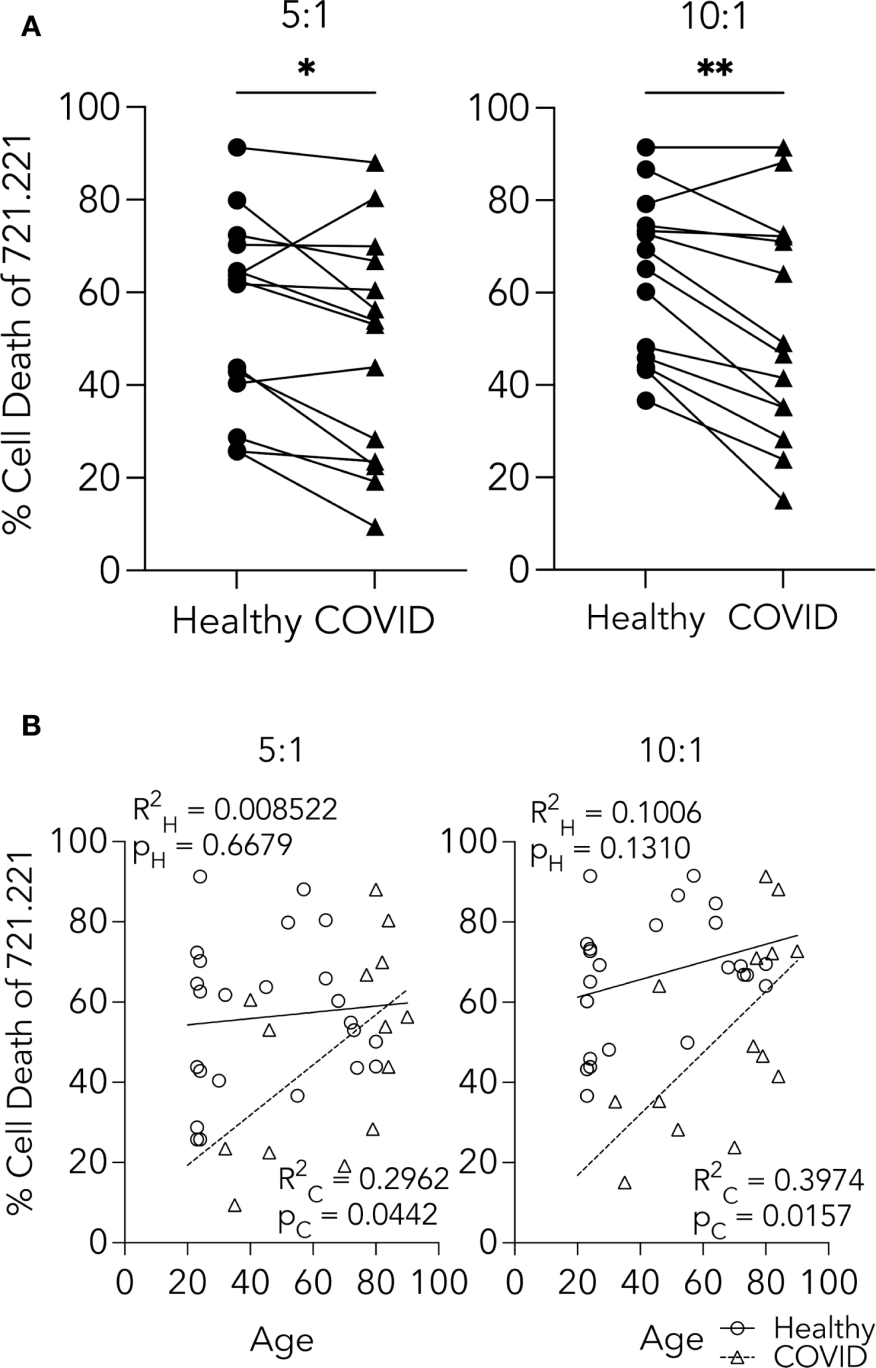
CD56+ CD3- NK cells from PHWC-19 have decreased cytotoxic abilities. **(A)** NK cell killing of 721.221 tumor cells is greater in healthy individuals than in PHWC-19. 721.221 cells were stained with CFSE to differentiate from the primary NK cells (pNKs). Cell death was measured using 7AAD using flow cytometry. The line connecting the healthy and COVID-19 points were paired samples obtained and conducted simultaneously across two E:T ratios of 5:1 and 10:1. Each point is a representative average of a triplicate result. N = 14. **(B)** Age-related comparisons of NK cell killing between the healthy cohort and PHWC-19 at two E:T ratios (5:1 and 10:1). Circles indicate NK cells from healthy individuals, while triangles indicate PHWC-19. * = P<0.05, ** = P<0.01, *** = P<0.001; calculated using the paired T-test.

To test the cytotoxic ability of NK cells from PHWC-19, we used an established method of tumor target killing (Epling-Burnette et al., 2007; Lisovsky et al., 2015; MacFarlane et al., 2017). NK cells were co-cultured with the 721.221 cell line, and killing was assessed using 7-AAD uptake and flow cytometry. There was a decrease in 721.221 cell death in response to NK cells from PHWC-19 at E:T ratios of 5:1 and 10:1 compared to healthy control subjects (**Figure 2A**).

Since the mean age of PHWC-19 was older than that of our healthy control subjects, and aging has been reported to affect some aspects of immune cell function, we tested the effect of age on NK cell cytotoxicity (Solana and Mariani, 2000; Solana et al., 2006). Linear regression analysis of the healthy donors showed no decline in the percentage of 721.221 cells killed when co-cultured with NK cells from older healthy subjects (**Figure 2B**). Surprisingly, there was a significant increase in the percentage of 721.221 cells killed with increasing age of the PHWC-19 (**Figure 2B**). Although there were fewer young PHWC-19, results were significant, revealing that COVID-19 caused a defect in cytotoxicity that was greater in younger patients than in older patients who required hospitalization.

### NK cell effector molecules and degranulation were altered in PHWC-19

**Figure 3:**
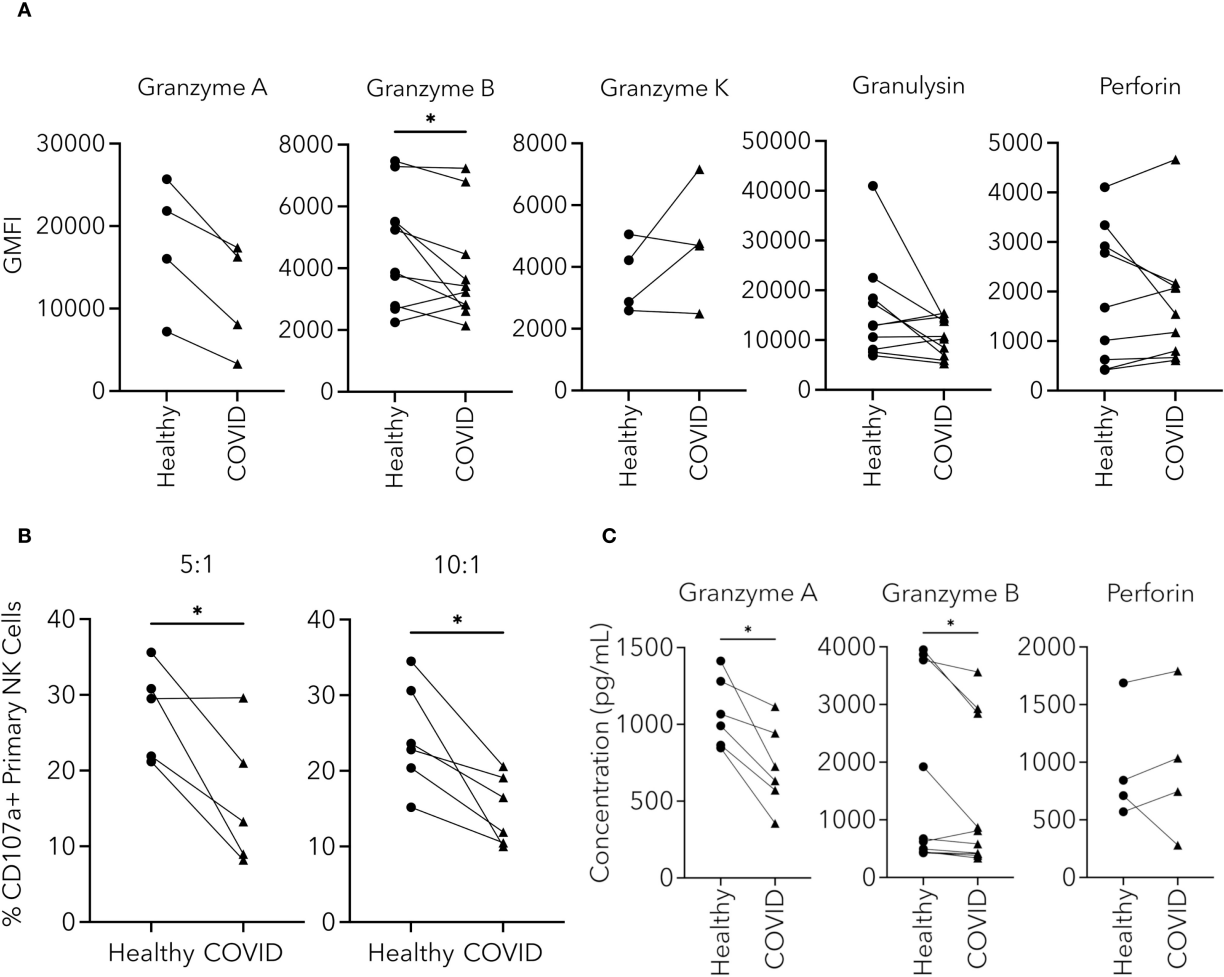
NK cell effector molecules and degranulation were altered in NK cells from PHWC-19. **(A)** Changes in the levels of intracellular effector molecules within CD56+CD3- cells from the blood of healthy individuals and PHWC-19. The levels of intracellular effector molecules granzymes A (N = 4), B (N = 10), K (N = 4), perforin (N = 9), and granulysin (N = 10) were measured using fluorescent antibodies and detected by flow cytometry in simultaneous experiments. **(B)** NK cell degranulation is reduced in NK cells from PHWC-19 than in healthy patients. The levels of CD107a were labelled using fluorescent antibodies (anti-CD107a) after the NK cells were co-cultured with 721.221 tumor cells at various E:T ratios and detected by flow cytometry. 5:1 (N = 5) and 10:1 (N = 6). **(C)** Changes in the concentration of effector molecules released from NK cells from healthy individuals and PHWC-19. The granzyme A (N = 6), granzyme B (N = 10), and perforin (N = 4) levels in the supernatants collected from a co-culture of the pNKs with 721.221 tumor cells were detected using ELISA. Experiments were conducted at an E:T ratio = 10:1. Circles indicate healthy individuals, while triangles indicate PHWC-19. * = P<0.05, ** = P<0.01, *** = P<0.001. Statistics for A and C were calculated using the Wilcoxon matched-pairs signed rank test; statistics for B were calculated using the paired T-test after testing for a normal distribution.

After establishing that cytotoxicity was reduced in NK cells from PHWC-19, we were interested in the sequence of intracellular events that might account for the reduced cytotoxicity. Effector molecules are preformed and stored in granules before their release. Granzymes A, B, and K are serine proteases, while perforin and granulysin are membrane-disrupting or pore-forming proteins involved in the defense against tumors and viruses (Krensky and Clayberger, 2009; van Domselaar and Bovenschen, 2011; Thiery and Lieberman, 2014). Using intracellular antibody labelling and flow cytometry, we compared the relative abundance of intracellular granzymes A, B, K, perforin and granulysin in NK cells from PHWC-19 and healthy adults. We found that only the intracellular levels of granzyme B were significantly decreased in the NK cells of PHWC-19 compared to those from healthy subjects (**Figure 3A**), and the fluorescent intensity of granzyme B correlated with cytotoxicity (**Supplementary Figure 2A**), revealing that the level of granzyme B was important for cytotoxicity. Intracellular granzyme A showed a lower trend in NK cells from PHWC-19 but did not reach statistical significance using a Wilcoxon matched-pairs signed rank test (**Figure 3A**).

In contrast, granzyme K showed no significant differences between the healthy and COVID-19 groups (**Figure 3A**). Among the pore-forming effector molecules, intracellular perforin and granulysin were not significantly different between the healthy and COVID-19 samples (**Figure 3A**). The intracellular content of granzymes and pore-forming proteins reflects the stores available. However, since only a fraction of the stores are utilized during each round of killing, the effect would potentially manifest only after multiple rounds of killing, leading to the depletion of the stores (Gwalani and Orange, 2018). Because of this, we were interested in whether degranulation was affected.

Degranulation occurs when the effector-containing granules fuse with the NK cell plasma membrane and release their contents. To assess degranulation in NK cells from patients hospitalized with COVID-19, flow cytometry was used to detect the cell surface membrane expression of CD107a (LAMP1) during the killing of tumor cells. There was a significantly lower expression of CD107a after co-culture of NK cells from PHWC-19 with 721.221 cells, and the fluorescent intensity of CD107a correlated with cytotoxicity (**Supplementary Figure 2B**), revealing the importance of reduced degranulation of NK cells from hospitalized SARS-CoV-2 infected patients compared to healthy NK cells (**Figure 3B**).

The release of effector proteins during degranulation increases the concentration of effectors in the extracellular compartment. Since degranulation was reduced in NK cells from patients hospitalized with COVID-19, we then determined if there was a difference in the amount of effector molecules released. The supernatants of the co-cultures were collected, and the effector molecules, granzyme A, B, and perforin, were measured by ELISA. There was a significant decrease in the concentration of granzymes A and B released from the NK cells obtained from PHWC-19 (**Figure 3C**). Meanwhile, extracellular concentrations of perforin were not significantly different between the healthy and COVID-19 cohorts (**Figure 3C**). This data reveals that intracellular stores of critical effector molecules were reduced, and degranulation and the release of granzymes were impaired in NK cells from PHWC-19, which was associated with reduced cytotoxicity.

### Granule trafficking was impaired in NK cells from PHWC-19

**Figure 4:**
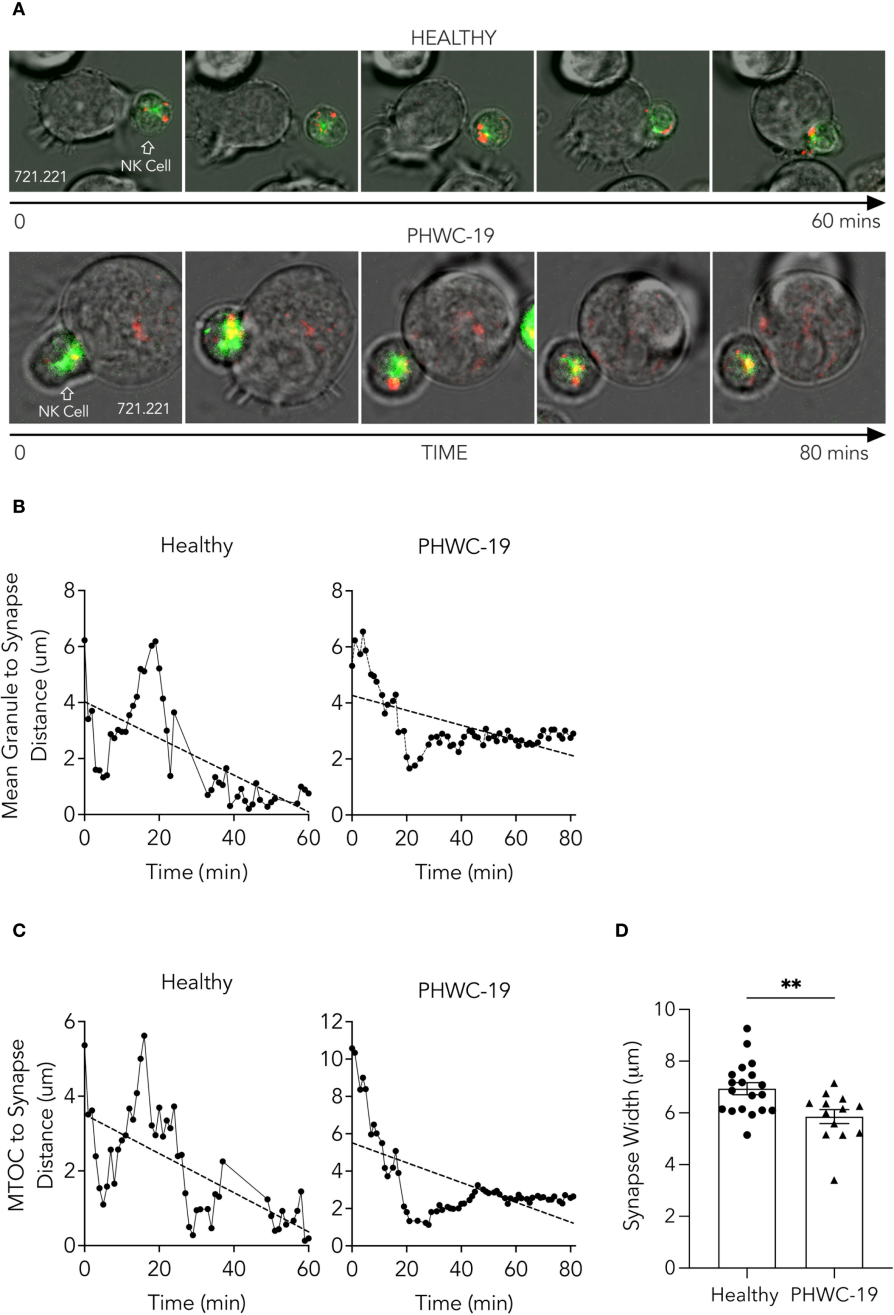
Trafficking of NK cell granules and the MTOC towards the synapse was impaired in NK cells from PHWC-19. **(A)** NK cells were incubated with 721.221 tumor cells and stained with Cresyl violet (granules-red) and Tubulin tracker green (microtubules-green). Cells were imaged for 60–80 minutes at a magnification of 60x. Images correspond to **Supplementary Video 1A and 1B**. **(B)** The distance between the mean of the granules and the synapse (μm) in NK cells in patients hospitalized with COVID-19. **(C)** The distance between the microtubule organizing center (MTOC) and the synapse (μm) in NK cells from patients hospitalized with COVID-19. The distances were measured using ImageJ software. N_Healthy_=3. N_COVID_=3. **(D)** The width of the synapse was determined when the plasma membrane of the NK cell and the tumor cell was deformed by the contact and defined as the distance between the points of contact of both cells’ plasma membranes (μm). N = 19 conjugates for healthy donors and N = 13 conjugates for PHWC-19. P<0.05. Statistics for D were calculated using an unpaired T-test after testing for a normal distribution.

To release the effector molecules at the target cell interface, the intracellular cytolytic granules must move along the microtubules and reach the area of contact with the target cell. We sought to determine if there was impaired trafficking of the granules and the microtubule-organizing center (MTOC). Granule trafficking requires the granules to converge on the MTOC via microtubule motor proteins and for the converged granules and MTOC to polarize to the NK cell synapse with the target cell (Ogbomo et al., 2018). Since enveloped viruses disrupt many forms of cellular transport (Hassan et al., 2021), we asked whether the defect in granule release was due to deficiencies in trafficking or failure to reach the plasma membrane. To examine granule movement, we used live-cell imaging of NK cells from healthy subjects and PHWC-19 in the presence of 721.221 target cells. NK cells from healthy subjects rapidly mobilized the granules and MTOC to be adjacent to the tumor cell, indicating successful granule trafficking (**Figure 4A**, **Supplementary Video 1A**). By contrast, the cytolytic granules and MTOC in NK cells from PHWC-19 did not polarize to the synapse with the target cell, indicating failed granule trafficking, which would lead to defective granule release and cytotoxicity (**Figure 4A**, **Supplementary Video 1B**). The imaging of NK cells from hospitalized patients with COVID-19 was continued for an additional 20 minutes to capture any events that may have taken longer to occur. Quantifying the mean granule-to-synapse distance indicated decreased localization of granules towards the synapse in NK cells from PHWC-19 (**Figure 4B**), although the time to initial polarization was similar in healthy and PHWC-19 (**Supplementary Figure 3**). Similarly, reduced localization of the MTOC was also seen in the NK cells of PHWC-19 (**Figure 4C**).

NK cells from healthy subjects formed a broad, concave base of attachment between the NK cell and the tumor cell. By contrast, the NK cells from PHWC-19 formed a synapse with the tumor cells, but the synapse was not as intimate as the synapse formed by NK cells from healthy control subjects, and the width of the synapse was greater in NK cells from healthy control subjects than in NK cells from PHWC-19 (**Figures 4A, D**, **Supplementary Video 1B**). Thus, NK cells from PHWC-19 demonstrated impaired granule trafficking and a less intimate synapse formation that would contribute to defective cytotoxicity.

### Expression of inhibitory and activating NK cell surface receptors

**Figure 5:**
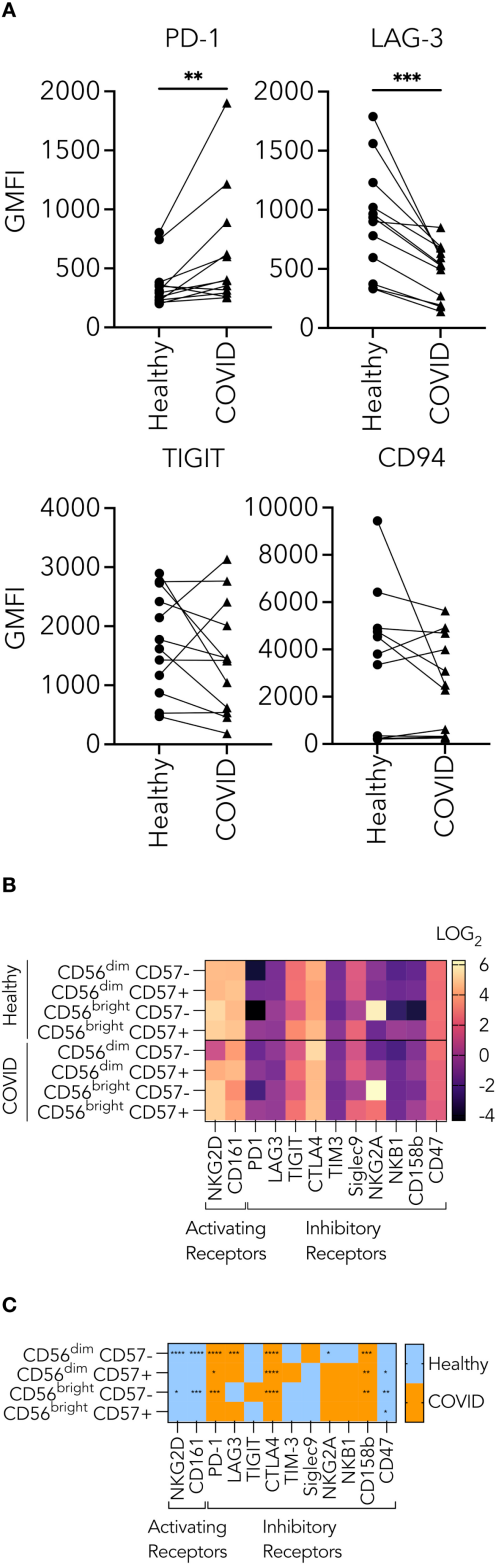
Receptors expressed on the surface of natural killer cells from healthy subjects and PHWC-19. **(A)** Changes in the levels of checkpoint inhibitors on CD56+ CD3- PBMCs from healthy individuals and PHWC-19 in paired experiments. Triangles indicate PHWC-19, while circles indicate simultaneous healthy individuals. The levels of checkpoint inhibitors, LAG-3 (N = 12), PD-1 (N = 6), TIGIT (N = 12), and CD94 (N = 10) were labelled using fluorescent antibodies and detected by flow cytometry. **(B)** Heatmap of log_2_ expression of activating and inhibitory receptors in NK cell subsets from whole blood of healthy donors (N = 15) and patients hospitalized due to COVID-19 (N = 17) assessed by single-cell mass cytometry. Lighter colors (larger numbers) on the legend indicate greater receptor expression than the darker colors (smaller numbers). **(C)** The difference between the healthy and COVID cohorts in the expression of activating and inhibitory receptors in NK cell subsets from whole blood of healthy donors (N = 15) and PHWC-19 (N = 17). Blue indicates higher expression in healthy donors than in PHWC-19, while orange indicates higher expression in PHWC-19 than in healthy donors. $\left(Difference=Mean_{Healthy}-Mean_{COVID}\right)$ . Data was collected by single-cell mass cytometry. * = P<0.05, ** = P<0.01, *** = P<0.001. Statistics for A were calculated using the Wilcoxon matched-pairs signed rank test; statistics for C were calculated using the Mann-Whitney test.

Because of the suboptimal conjugate formation and granule polarization in NK cells from PHWC-19, we hypothesized that there might be changes in inhibitory receptor expression, which could affect granule trafficking and degranulation. The inhibitory receptors include a subset called checkpoint inhibitory proteins. These proteins regulate the NK cell function by reducing their cytolytic activity. We performed experiments using samples of NK cells obtained from healthy and PHWC-19 on the same day to assess PD-1, LAG-3, TIGIT, and CD94. Only PD-1 was significantly increased in NK cells from PHWC-19 (**Figure 5A**). Meanwhile, LAG-3 expression was reduced in PHWC-19, and TIGIT and CD94 showed no difference between the healthy and COVID-19 groups (**Figure 5A**). Since different cell subsets are present, we examined the expression patterns of inhibitory and activating receptors on different NK cell subsets defined by CD56 and CD57 expression. Using mass cytometry, we studied the expression of the inhibitory and checkpoint receptors PD-1, LAG-3, TIGIT, CTLA4, TIM-3, Siglec 9, NKG2A, NKB1, CD161, CD158b, CD47 and the activating receptor NKG2D on these subsets. The differential expression of each receptor within the subsets was visualized via a log2 heatmap (**Figure 5B**). The more mature and most cytotoxic CD56^dim^ CD57+ NK cells in PHWC-19 showed a higher expression of PD-1, CTLA-4, and CD158b and a decrease in CD47 compared to healthy controls, suggesting that the net effect is to dampen cytotoxicity (**Figure 5C**). Similarly, the more abundant and slightly less cytotoxic CD56^dim^ CD57- NK cells showed a significant upregulation of PD-1, LAG3, CTLA4, and CD158b, along with a downregulation of NKG2D, NKG2A, and CD161 in PHWC-19 compared to healthy controls. This also suggests a net negative effect (**Figure 5C**). Overall, these cytotoxic NK cell populations exhibit an increased expression of inhibitory receptors in PHWC-19, which is consistent with reduced killing. In the less prevalent CD56^bright^ CD57- NK cells, which are associated with cytokine production and lower cytotoxicity, there was an increased expression of PD-1, CTLA4, CD158b, and decreased expression of NKG2D, CD161, and CD47 in PHWC-19 (**Figure 5C**), revealing a receptor profile consistent with a negative effect on signaling.

**Figure 6:**
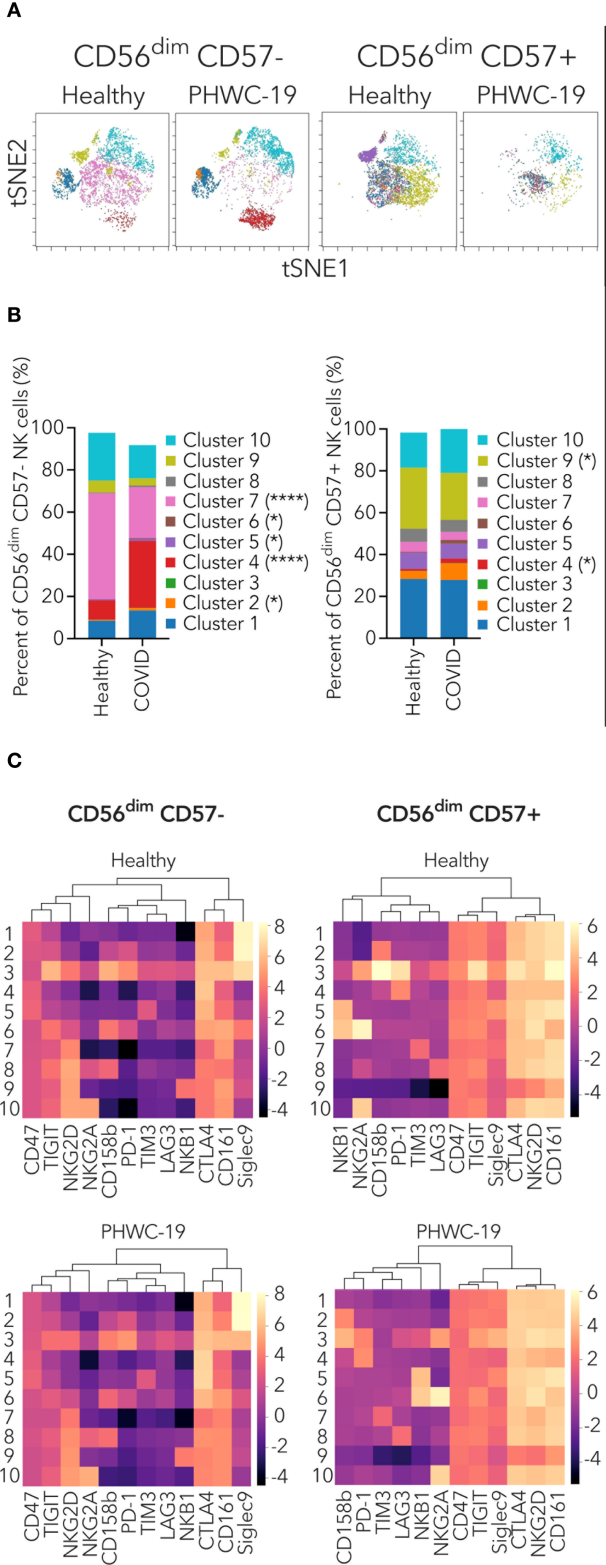
Mass cytometry profiling of NK receptor co-expression on specific CD56^dim^ NK cell populations. **(A)** Representative t-SNE dimensionality reduction plots of the clusters within the CD56^dim^ CD57- (left) and CD56^dim^ CD57+ (right) NK cell populations from healthy donors (N = 15) and PHWC-19 (N = 17). **(B)** Pooled relative percentages of each cluster within the CD56^dim^ CD57- (left) and CD56^dim^ CD57+ (right) NK cell populations from healthy donors (N = 15) and PHWC-19 (N = 17). The legend applies to sections **(A, B, C)** Heatmap and dendrogram of log_2_ expression of activating and inhibitory receptors within the clusters of CD56^dim^ NK cell subset from whole blood of healthy donors (N = 15) and PHWC-19 (N = 17). Data was collected by single-cell mass cytometry. Lighter colors (larger numbers) on the legend indicate greater receptor expression than the darker colors (smaller numbers). Hierarchical clustering heat map was drawn using ChiPlot (https://www.chiplot.online/). * = P<0.05, ** = P<0.01, *** = P<0.001. Statistics for B were calculated using the Mann-Whitney test.

We identified major clusters within CD56^dim^ CD57- and CD56^dim^ CD57+ NK cells based on the co-expression of activating and inhibitory receptors (**Figure 6A**). This aided us in identifying alterations in NK cells with specific receptor co-expression between healthy donors and PHWC-19. The major clusters for CD56dim CD57- NK cells were 1, 4, 7, 9, and 10 (**Figure 6B**). Among these, cluster 4 was significantly increased in PHWC-19, while cluster 7 was significantly decreased in PHWC-19 (**Figure 6B**). Cluster 4 of CD56^dim^ CD57- NK cells was characterized by higher co-expression of CTLA4, CD161, and CD47, while cluster 7 showed higher co-expression of NKG2D, CTLA4, and CD161 (**Figure 6C**). Dendrogram clustering revealed three major groups of receptors co-expressed on CD56^dim^ CD57- NK cells, which showed similarities between healthy and PHWC-19 groups (**Figure 6C**).

The major clusters for CD56dim CD57+ NK cells were clusters 1, 9, and 10 (**Figure 6B**). Cluster 9 was significantly decreased in PHWC-19 (**Figure 6B**). This cluster showed higher co-expression of NKG2D, TIGIT, CTLA4, CD161, and CD47 (**Figure 6C**). Dendrogram clustering revealed four major groups of receptors co-expressed on CD56^dim^ CD57+ NK cells, which were similar between healthy and PHWC-19 donors (**Figure 6C**). We also investigated the clusters within CD56^bright^ CD57- and CD56^bright^ CD57+ NK cells, but these populations did not show major differences between healthy donors and PHWC-19 (**Supplementary Figures 4A–C**). The dendrograms of CD56^bright^ CD57- and CD56^bright^ CD57+ NK cells showed similar groupings in the healthy and PHWC-19 groups (**Supplementary Figure 4C**). Additional PCA analysis was performed on CD56^bright^ and ^dim^ and CD57+ and – subsets (**Supplementary Figure 5**). These results reveal that specific combinations of inhibitory receptors cluster into major subsets and that these clusters differ in their expression in PHWC-19 compared to control subjects.

### Effect of variants, dexamethasone, sex, and vaccine status on NK cell killing in patients hospitalized with COVID-19

**Figure 7:**
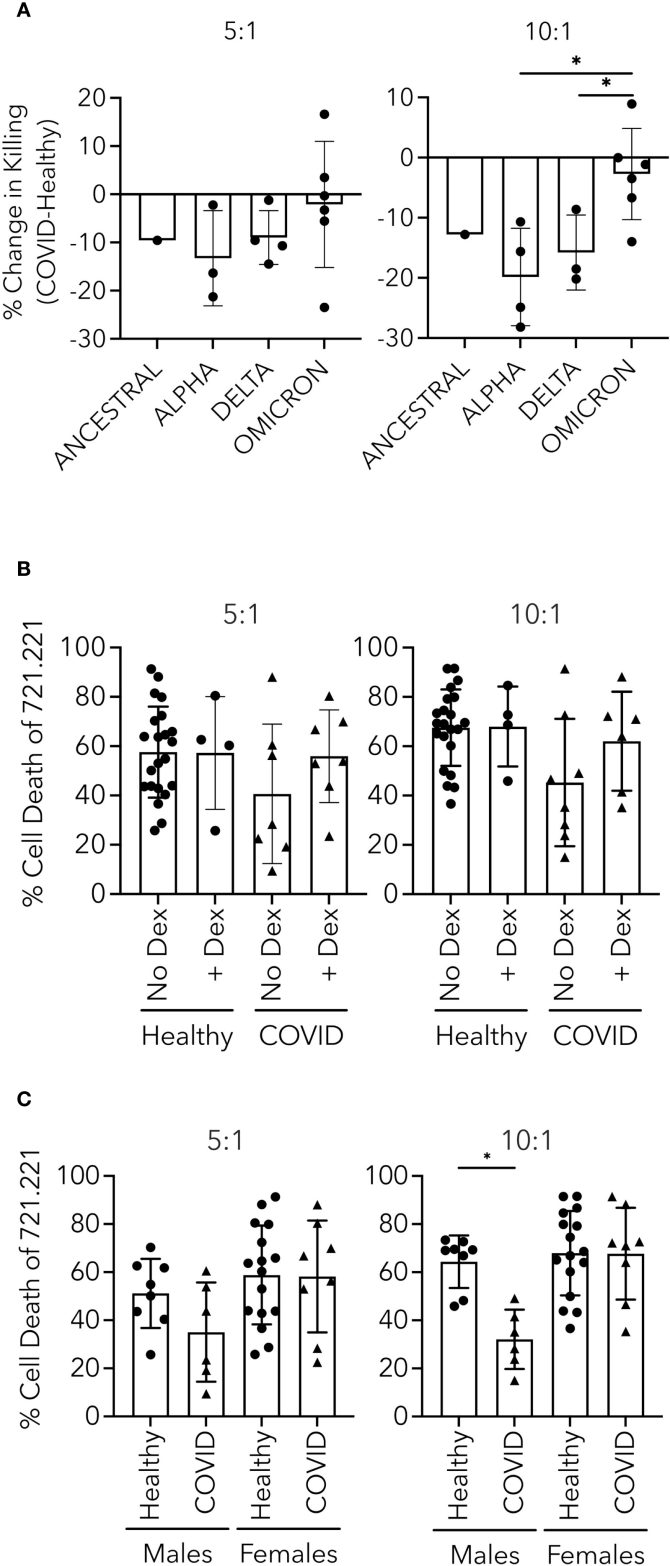
Effect of variant, dexamethasone and sex on NK cell killing in PHWC-19. **(A)** 721.221 tumor cell killing by NK cells from PHWC-19 improved with later SARS-CoV-2 variants. Each point indicates the change in killing ability by subtracting the results of killing assays from paired healthy donors from the results from hospitalized patients with COVID-19 (N = 28). **(B)** Comparison of 721.221 tumor cell killing by NK cells from patients infected with COVID-19 receiving or not receiving dexamethasone. Circles indicate healthy donors, and triangles indicate patients hospitalized with COVID-19. To determine the effect of dexamethasone on NK cells from healthy subjects, dexamethasone was added to the cultured media (0.1 μM) (N_Healthy_=24 and N_COVID_=14). **(C)** NK cell killing of 721.221 tumor cells is more prominent in healthy males than hospitalized males with COVID-19. Circles indicate individual healthy donors, and triangles indicate individual patients hospitalized with COVID-19. Experiments were conducted at two effector-to-target ratios (E:T): 5:1 and 10:1. Each point represents a triplicate result’s average. Healthy males (N = 16), COVID males (N = 12), healthy females (N = 29), and COVID females (N = 16). * = P<0.05, ** = P<0.01, *** = P<0.001; calculated using the Kruskal-Wallis ANOVA with Dunn’s multiple comparisons test.

After establishing the intracellular mechanistic defects responsible for impaired cytotoxicity in PHWC-19, we wondered how other biological, therapeutic and virologic factors might influence the defect. These characteristics include the different variants of the SARS-CoV-2 virus, the treatment of patients with dexamethasone, and the sex-related differences. The SARS-CoV-2 virus underwent genetic mutations, producing variants of concern. The severity of the disease and immune responses differed depending on the variant causing the infection (Lin et al., 2021; Arabi et al., 2023). The major variants of concern sequentially evolved from Alpha to Beta, Gamma, Delta, and Omicron. These variants have shown increased transmissibility, decreased virulence, and antigenicity (defined by antibody binding) (Alefishat et al., 2022). We sought to determine the cytotoxicity of NK cells obtained from PHWC-19 infected with different SARS-CoV-2 variants compared to healthy control subjects. Unfortunately, the sample size was small, and while the differences in the degree of impaired NK cell killing did not reach statistical significance, it is worth noting that there is a trend suggesting that the reduction in NK cell cytotoxicity was greater with the Alpha variant compared to NK cells from PHWC-19 infected with Delta and Omicron (**Figure 7A**), which correlates with the lack of effective host defense and worsened pathology in patients infected with earlier variants of concern.

During the transition from the time when the Alpha variant was dominant to the time when the Delta variant was dominant, dexamethasone was routinely added to therapy for the management of hypoxemic hospitalized patients (The Recovery Collaborative Group, 2021). It is possible that dexamethasone diminished the function and the number of NK cells (Chen et al., 2018). To address this possibility, we performed experiments to determine whether dexamethasone affected cytotoxicity in PHWC-19. In these experiments, multiple comparisons were made. Control PHWC-19 who were enrolled before the use of dexamethasone or where the care team chose not to use it served as the comparator for PHWC-19 that received dexamethasone, and NK cells from healthy subjects were compared to their NK cells treated *in vitro* with dexamethasone. Previous studies suggested that the serum concentration after a dose of 6mg would be 0.1μM (Weijtens et al., 1998; Spoorenberg et al., 2014; Morgan and Davis, 2017). To evaluate the effect of the equivalent of 6mg dexamethasone on NK cells *in vivo*, NK cells from healthy control subjects were pretreated with 0.1 μM *in vitro*. There was no significant reduction in NK cell cytotoxicity between the NK cells from PHWC-19 treated or not treated with dexamethasone, and no reduction in cytotoxicity among healthy subjects treated with dexamethasone compared to the control *in vitro* (**Figure 7B**).

Differences in biological sex influence many aspects of the immune system, and we are beginning to learn their effect on infections. Males were more likely to become hospitalized when infected with SARS-CoV-2, and males comprise the majority of all age and ethnic groups in hospitalized patients (Qi et al., 2021). To determine whether biological sex affected NK cell cytotoxicity, the responses from NK cells from male PHWC-19 were compared to those of healthy male control subjects, and responses of NK cells from female PHWC-19 were compared to those of healthy female control subjects. There was a significant decrease in the killing of NK cells in male PHWC-19 compared to the healthy male subjects (**Figure 7C**). By contrast, we did not detect a difference in NK cell cytotoxicity in female PHWC-19 compared to healthy control females.

A multivariate analysis was performed to examine the influence of age, healthy vs PHWC-19, the influence of dexamethasone, and sex. The regression formulation for an E:T ratio of 10:1 was Cytotoxicity = 31.898 + 0.340*Age + 22.722*Status (healthy) + 9.143*Dexamethasone - 14.046*Sex (males). Where P = 0.010 (Age), 0.001 (Status-healthy), 0.015 (Sex-Male).

To determine whether vaccine status affected NK cell function in patients who had been hospitalized with COVID-19, the cytotoxicity was compared between NK cells from PHWC-19 who had been vaccinated and those who had not. This demonstrated no difference in the cytotoxicity (**Supplementary Figure 6**), suggesting that once hospitalized with COVID-19, vaccine status did not influence the reduction in NK cell function.

### Assessment of sex differences in cytotoxic mechanisms in healthy and hospitalized patients positive for COVID-19

Having shown that the defects in cytotoxicity resulting from COVID-19 disease were correlated with the male but not female sex, we were interested in exploring whether the intracellular defects also exhibited a similar pattern of sex-related differences. Unfortunately, the sample size was small, and while trends emerged, the intracellular expression of effector molecules, degranulation or extracellular free effector molecules released into the supernatant failed to demonstrate statistically significant differences between males and females (**Supplementary Figures 7A–C**).

**Figure 8:**
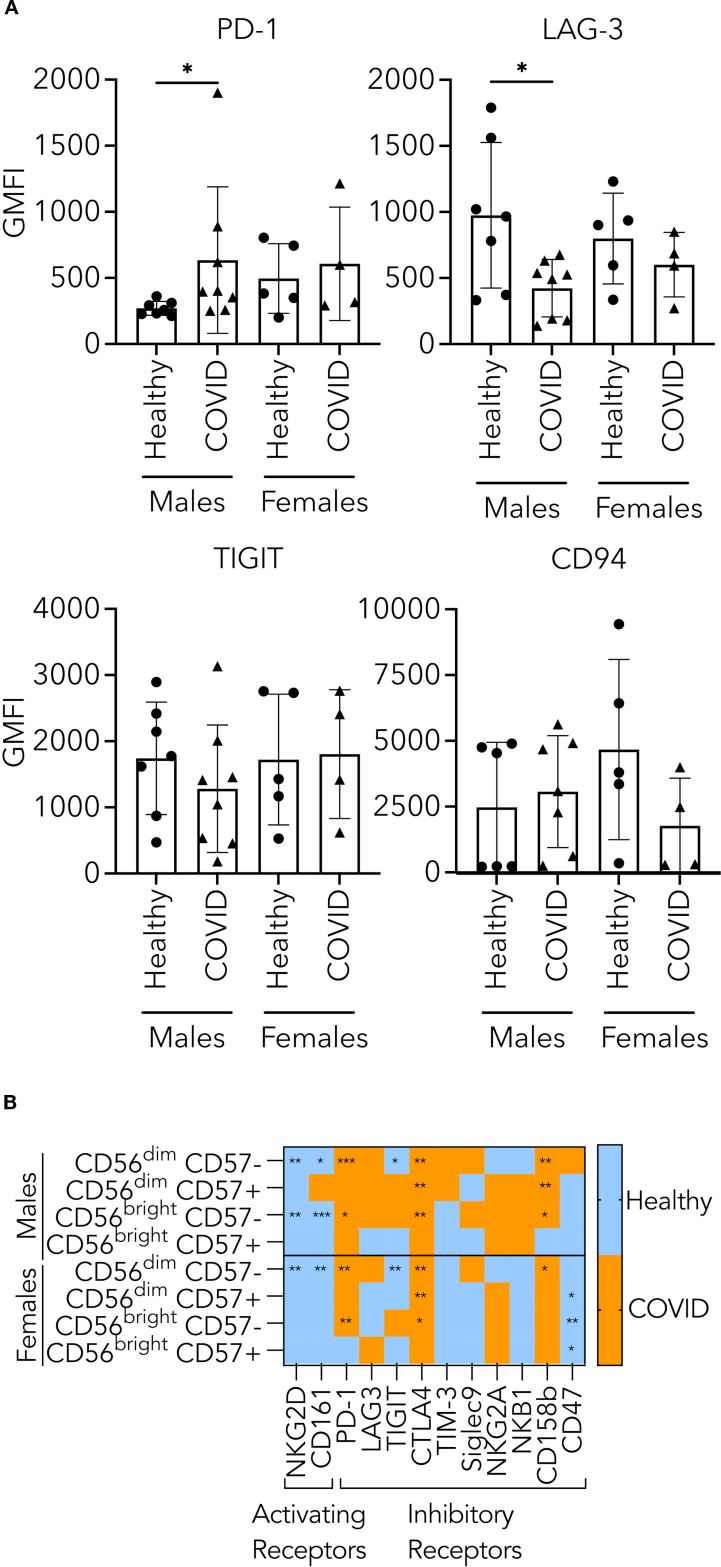
Assessment of sex differences in expression of activating and inhibitory receptors in healthy and hospitalized patients positive for COVID-19. **(A)** Comparison of the levels of checkpoint inhibitors on CD56+ CD3- PBMCs from healthy individuals and hospitalized patients who tested positive for COVID-19 for differences between sexes. The levels of checkpoint inhibitors like LAG-3 (N = 12), PD-1 (N = 6), TIGIT (N = 12), and CD94 (N = 10) were tagged using fluorescent antibodies and detected by flow cytometry. Circles indicate healthy donors, while triangles indicate patients hospitalized with COVID-19. **(B)** Comparison of the expression of receptors within NK cell subsets from healthy individuals (N = 15) and PHWC-19 (N = 17) for differences between sexes. Receptor expression was measured using lanthanide-labelled antibodies and detected by mass cytometry. Blue indicates higher expression in healthy donors than in PHWC-19, while orange indicates higher expression in PHWC-19 than in healthy donors. $\left(Difference=Mean_{Healthy}-Mean_{COVID}\right)$ . * = P<0.05, ** = P<0.01, *** = P<0.001. Statistics for A were calculated using Kruskal-Wallis ANOVA with Dunn’s multiple comparisons test; statistics for B were calculated using the Mann-Whitney test.

We also assessed the expression of activating and inhibitory receptors in male versus female participants to determine whether there were differences. NK cells from male PHWC-19 showed an increased expression of PD-1 compared to healthy subjects, while females did not (**Figure 8A**). By contrast, there were no differences in TIGIT and CD94 expression in males or females (**Figure 8A**).

Further examination of the NK cell subsets revealed similar changes in males and females, including upregulation of PD-1, CTLA4, and CD158b, compared to sex-matched controls for the CD56^dim^ CD57- NK cells (**Figure 8B**). Meanwhile, CD56^dim^ CD57+ NK cells from male PHWC-19 showed a higher expression of CTLA4 and CD158b and NK cells from female PHWC-19 had a higher expression of CTLA4, which was balanced by a lowered CD47 expression (**Figure 8B**). Lastly, in the CD56^bright^ CD57- NK cells, male PHWC-19 exhibited more of an inhibitory phenotype with increased CD158b and decreased NKG2D expression (**Figure 8B**). In females, more inhibitory receptors are expressed in the healthy donors or evenly expressed between the healthy and PHWC-19 cohorts (**Figure 8B**). In summary, there were significant differences in the male patients, including reduced activating receptor expression and increased inhibitory receptors.

Two studies have shown the sexually dimorphic nature of dexamethasone treatment that is more beneficial in men than in women (The Recovery Collaborative Group, 2021; Sinha et al., 2022). We were interested in whether this effect extended beyond neutrophils and involved NK cells. While there was no difference at a 5:1 ratio, at the 10:1 ratio, males who did not receive dexamethasone had a trend towards a more pronounced NK cell cytotoxicity defect than male PHWC-19 who received dexamethasone (**Supplementary Figure 8**). While dexamethasone treatment may not show overall effects on NK cell function, dexamethasone may have a role in recovering NK cell cytotoxicity specific to males.

## Discussion

NK cells are crucial in defending against viral infections, including killing SARS-CoV-2-infected cells. While other viruses affect NK cell responses, gaps remain in understanding NK cell function in SARS-CoV-2-infected patients. Most studies enrolled severe hospitalized COVID-19 cases; our study focused on patients with moderate disease not requiring ICU care. Few studies have explored NK cell function across different COVID-19 variants, vaccination statuses, and dexamethasone use. Studies have not previously investigated the sequence of events leading to NK cell cytotoxicity to completely understand how the infection changes NK cell responses over time. Our study is novel in examining the interactions between receptor subset clustering, stores of effector molecules, granule trafficking, polarization of granules, and effector molecule release in the context of a viral infection, and the roles of sex and dexamethasone on NK cell cytotoxicity and mechanisms. Our research will drive NK cell-dependent therapies for PHWC-19 by targeting the possible dysfunctions we outlined (Hernández-Blanco et al., 2025).

Our study builds upon previous work in the field, showing a reduction in the percentages of all mononuclear cells and, more specifically, a reduction in the proportion of NK cells from patients hospitalized with COVID-19 (Zheng et al., 2020; Hermens and Kesmir, 2023; Mansourabadi et al., 2023). We also found that CD56^dim^ NK cells comprise most NK cells, and the ratio of CD56^dim^ to CD56^bright^ was preserved in PHWC-19 (Fan et al., 2008). However, our studies revealed that CD56^dim^ CD57- NK cells were increased in PHWC-19, while CD56^dim^ CD57+ cells were reduced. This change contrasts with HIV, in which CD57- cells are reduced, while the number of CD57+ cells remains the same (Hong et al., 2010). In addition to reducing the number of NK cells, it indicates a shift to a more immature, less cytotoxic phenotype in PHWC-19 that could contribute to the observed defect in NK cell cytotoxicity (Lopez-Vergès et al., 2010).

While the number of NK cells is important, their cytotoxic function is crucial to maintaining host defense. Previous studies showed reduced NK cell degranulation in severe COVID-19 patients against K562 and SW480 tumor cells but did not assess cytotoxicity or moderately ill patients (Krämer et al., 2021; Varchetta et al., 2021). Reduced CD56^dim^ CD57+ NK cells may impair cytotoxicity, as they are more cytotoxic than increased immature CD56^dim^ CD57- NK cells (Nielsen et al., 2013).

We examined age-related changes, noting that although the average age of the COVID-19 group was older, younger PHWC-19 showed a relatively greater defect in NK cell cytotoxicity. Increasing age did not adversely affect NK cell cytotoxicity in either cohort. Surprisingly, advanced age was associated with improved NK cell function in PHWC-19, consistent with studies linking NK cell function more to health status and comorbidities than age (Solana and Mariani, 2000; Ogata et al., 2001; Solana et al., 2006). The increase in NK cell function was even more apparent in healthy control subjects, making it unlikely that inflammation or NK cell memory contributed to the increase. We further investigated the mechanisms behind the impaired NK cell cytotoxicity.

We began investigating NK cell processes sequentially by examining the effector molecules and granule-mediated release. NK cells deploy stored lytic granules to release the effector molecules during cytotoxic responses. Granzymes and pore-forming effector molecules are stored within the NK cell and, when needed, are released upon activation. Perforin is crucial in helping granzymes enter target cells (Thiery and Lieberman, 2014). Without perforin, granzymes are less effective at entering target cells, directly affecting cytotoxicity. While some studies indicate elevated perforin in severe COVID-19 patients, others have reported reduced or unchanged levels (Jiang et al., 2020; Maucourant et al., 2020; Mazzoni et al., 2020; Bozzano et al., 2021; Witkowski et al., 2021; Zenarruzabeitia et al., 2021). Our findings align with the latter, showing no difference in perforin levels between healthy and COVID-19 groups, possibly due to differences in disease severity (Zenarruzabeitia et al., 2021).

We observed an overall reduction in granzyme B in NK cells taken from moderate illness patients, which reflects findings in patients with severe illness from COVID-19 (Jiang et al., 2020; Zheng et al., 2020). We extended these findings by showing lowered degranulation and extracellular concentrations of granzyme B in the supernatant post-stimulation. By contrast, other reports observed an early upregulation of granzyme B, primarily evident in patients with severe COVID-19 (Maucourant et al., 2020; Witkowski et al., 2021; Zenarruzabeitia et al., 2021). Though less studied, granzyme A is critical for NK cell cytotoxic function (Grossman et al., 2004; Chowdhury and Lieberman, 2008; van Domselaar and Bovenschen, 2011). Previous studies noted increased granzyme A+ NK cells in PHWC-19 but did not measure extracellular levels (Jiang et al., 2020; Li et al., 2020; Mazzoni et al., 2020). Our study revealed a significant reduction in the extracellular concentrations of granzyme B and a trend toward a decrease in granzyme A from NK cells of PHWC-19. This discrepancy may depend on disease severity. We also demonstrated a positive correlation between intracellular granzyme B levels and cytotoxicity, which supports the idea that a reduction in granzyme B levels in PHWC-19 would contribute to decreased cytotoxic abilities of NK cells.

Intracellular effector molecule levels reflect cellular readiness, but assessing their release is crucial for understanding impaired cytotoxicity. We observed decreased degranulation of NK cells from PHWC-19 in response to 721.221 cells, and found a positive correlation between degranulation and the cytotoxic ability of NK cells, extending previous reports of reduced CD107a expression in response to K562 tumor cells in PHWC-19 (Zheng et al., 2020; Witkowski et al., 2021). Other viruses, such as influenza A virus, exhibited inhibition of granule release against tumor cells, while human T-lymphotropic virus-1 and human cytomegalovirus upregulate degranulation (Mao et al., 2010; Tschan-Plessl et al., 2016; Amorim et al., 2019). Extracellular levels of perforin and granzymes A and B revealed decreased granzyme release from PHWC-19 NK cells, contributing to reduced cytotoxicity, especially in line with reduced degranulation. However, no change was observed in extracellular levels of perforin, indicating that reduced degranulation does not always mean a reduction in effector molecule availability. Nevertheless, our findings led us to conclude that alterations in NK cell effector molecules occur at all three stages, including stores, degranulation and effector release.

Having demonstrated that the release of effector molecules was reduced, we were interested in determining whether a defect in the transport process failed to bring these molecules to the synapse with the target cell. Lytic granules containing effector molecules are mobilized along the NK cell microtubules to position them at the immunological synapse before release. We assessed conjugate formation with the target and polarization of granules and the MTOC to the synapse. The findings demonstrate that the initial phase of conjugation progresses normally while the later stages of granule polarization are impaired. This study extends previous observations of defective granule polarization in NK cells from HIV patients by demonstrating similar defects in NK cells from PHWC-19 (Kyei et al., 2016). Although certain tumors resist NK cell killing by preventing granule polarization despite proper conjugation, we found that conjugation was defective, as indicated by the width and morphology of the synapse (Eitler et al., 2021). This critical process explains why the cells may not degranulate or release effector proteins, even if their stores were sufficient.

Due to interrupted granule trafficking and impaired synapse formation in NK cells from PHWC-19, we examined whether receptor expression was affected, potentially influencing signaling and cytotoxic responses. NK cell subsets were predominantly skewed towards an inhibited phenotype based on NKG2D, PD-1, LAG3, CTLA4, and CD158b (KIR2DL2/DL3) expression in PHWC-19. Previous studies focused on decreased NKG2D and increased PD-1 expression across all NK cells rather than specific subsets (Varchetta et al., 2021; Lee et al., 2023; Ustiuzhanina et al., 2023). Our study also investigated LAG3 and CTLA-4, finding increased expression favoring an exhausted phenotype in PHWC-19 (Wilk et al., 2020; Zheng et al., 2020; Claus et al., 2023). We found significant increases in CD158b in immature cytotoxic CD56^dim^ CD57- NK cells and the immunoregulatory CD56^bright^ CD57- subset (Poli et al., 2009). These changes are similar to human cytomegalovirus, which showed an increase in inhibitory receptors and a reduction in the activating receptors (Béziat et al., 2011; Muntasell et al., 2013; Tschan-Plessl et al., 2016), but dissimilar to hepatitis C virus, which upregulates the expression of activating receptors, including NKG2D, while lowering the expression of the inhibitory receptor KIR3DL1 (Oliviero et al., 2009; Ahlenstiel et al., 2010). Different viruses will target different receptors to modulate the functioning of NK cells.

By examining inhibitory receptors, we found that subsets of NK cells are more likely to express various families of these receptors in healthy donors and PHWC-19, indicating that individual NK cells expressing a particular receptor are more likely to express another specific receptor. Our study showed that the receptor clusters were similar between healthy donors and PHWC-19 within each NK cell subset, revealing that the clusters were maintained throughout the disease process. However, we also saw that clusters differed between CD57- and CD57+ cell subsets, suggesting that the maturation process alters these clusters. While previous studies have not examined these specific receptors, clusters can form due to differentiation or viral ligand exposure (Held et al., 2003; Goodridge et al., 2015; Rocca et al., 2022). The latter concept states that NK cell diversity may result from interactions with more infected host cells as they mature (Goodridge et al., 2015).

After examining the mechanisms related to NK cell function, we investigated correlations between demographic and clinical factors and NK cell function in PHWC-19 across different VOCs: Ancestral, Alpha, Delta, and Omicron. While studies have reported that NK cell dysfunction correlates with disease severity, none have examined the impact of different VOCs (Zheng et al., 2020). Despite small sample sizes, we found less severe defects in NK cell function in patients infected with Omicron than Delta and Alpha. The Alpha variant, dominant when vaccine administration was low, potentially resulted in more severe NK defects than in vaccinated Omicron patients. Vaccine-induced antibody-dependent cellular cytotoxicity (ADCC) might compensate for classical pathway defects in NK cell cytotoxicity in Omicron infections (Hagemann et al., 2022). NK cells are capable of vaccine-induced ADCC, as seen in infections like HIV, malaria, and influenza (Haynes et al., 2012; Arora et al., 2018; Kavian et al., 2020). These observations may contribute to NK cell cytotoxic functions being less impaired in Omicron infections compared to Alpha and Delta (Patone et al., 2021; Wolter et al., 2022; Jacobs et al., 2023). Our study is limited in testing this hypothesis because we did not have adequate sample sizes across the various experiments.

Various interventions influencing immune responses were used throughout the pandemic, including vaccination and dexamethasone. We observed no difference in NK cell cytotoxicity between vaccinated and unvaccinated PHWC-19, suggesting that once hospitalized with COVID-19, vaccination status did not influence cytotoxicity. Alternatively, the responses of NK cells from patients who received dexamethasone were compared to those of patients who had not received dexamethasone and to healthy controls treated *in vitro* with serum concentrations equivalent to oral administration of 6 mg/day. Our findings confirm that defects in NK cell cytotoxicity in PHWC-19 are independent of dexamethasone administration (Witkowski et al., 2021). One study showed that dexamethasone impacts neutrophils in a sex-dependent manner, with males responding better than females during severe COVID-19 (The Recovery Collaborative Group, 2021; Sinha et al., 2022). Our study is the first to explore the sex-specific effects of dexamethasone on NK cell function. Previous research indicates that glucocorticosteroids like dexamethasone generally restore immune responses in males but do not improve them in females (Song et al., 2018; Farkouh et al., 2021). NK cells from male PHWC-19 were more responsive to dexamethasone, exhibiting a trend of less defective cytotoxicity than untreated males.

Our study on sex-related differences in NK cell cytotoxicity was motivated by findings that males tend to have more severe COVID-19 outcomes than females (Takahashi et al., 2020; Qi et al., 2021). We found a greater defect in NK cell cytotoxicity in males hospitalized with COVID-19 compared to females. Females undergo inactivation of a single X chromosome to balance gene expression from two X chromosomes (Lyon, 1962). Escaping X inactivation provides women with an advantage in tackling immune challenges because of higher expression levels of immunological factors encoded on the X chromosome, such as TLR7, TLR8, IL2R, CXCR3, and IRAK1 (Wang et al., 2016; Schurz et al., 2019). Similar patterns could be present during COVID-19, where women have shown an upregulation of genes regulating innate antiviral responses, TLR8, TLR9, and IFIT1-3 (Montaño Mendoza et al., 2023). This prompted further investigation into NK cell functional mechanisms. Despite small sample sizes, we observed differences in activating and inhibitory receptor expression. More inhibitory receptors are expressed in CD56^dim^ CD57+ and CD56^bright^ CD57- NK cells from male PHWC-19. These alterations might contribute to the cytotoxicity defect, but further studies are needed to understand the mechanisms behind these defects in male PHWC-19. Further studies would also be required to determine whether these results have implications for long COVID or post-viral remodeling.

COVID-19 disease, in addition to vaccine status and degree of exposure, as public health measures evolved, has continuously changed, making it hard to control extraneous factors. We aimed to collect and investigate various factors, but we were unable to control some elements, such as the timing in the hospital before the blood draw, which could influence NK cell responses, other medications like remdesivir and comorbidities. We tried to address the medications and comorbidities by excluding immunosuppressed individuals and those taking immunosuppressive drugs. Our study did not collect information to determine whether remdesivir influenced our results, although remdesivir has not been shown to affect NK cell functioning in isolation (Zhou et al., 2020; Cianci et al., 2023).

Our study found that NK cells in PHWC-19 have impaired cytotoxic function. We investigated several mechanisms related to NK cell cytotoxicity and discovered that receptor expression skews towards an inhibited phenotype and are co-expressed in specific clusters, possibly affecting granule polarization, effector molecule production, and degranulation. The defect stems from various concurrent malfunctions in mechanisms. Variant and sex-dependent differences were observed, but further studies are needed to understand the underlying mechanisms.

## Data Availability

The raw data supporting the conclusions of this article will be made available by the authors, without undue reservation.

## Supplementary material

Supplementary Figure 1 Gating strategy for mass cytometry. The HELIOS machine was used for acquisition, and the data was uploaded to the Cytobank program, where manual hierarchical gating was performed after barcoding and labelling with lanthanide–labelled antibodies. All cell populations were initially gated on CD45+ to identify white blood cells via a histogram. Cellular populations were further identified with the following definitions: B cells (CD45+ CD19+), NKT Cells (CD45+ CD3+ CD56+), CD8+ T cells (CD45+ CD3+ CD8+), CD4+ T cells (CD45+ CD3+ CD4+), NK cells (CD45+ NKp46+), and monocytes (CD45+ CD14+). NK cells were further stratified into subsets of CD56^dim^ CD57-, CD56^dim^ CD57+, CD56^bright^ CD57-, and CD56^bright^ CD57 +. No signal was detected below zero, which caused the abrupt cutoff.

Supplementary Figure 2 The correlation of granzyme B expression and degranulation with cytotoxicity. **(A)** 721.221 tumor cell killing by NK cells increases with increasing intracellular granzyme B levels in NK cells from healthy individuals and PHWC-19 (N_Healthy_=8 and N_COVID_=8) **(B)** 721.221 tumor cell killing by NK cells increases with increasing degranulation in NK cells from healthy individuals and PHWC-19 (N_Healthy_=10 and N_COVID_=10). Circles indicate healthy individuals, while triangles indicate PHWC-19. Experiments were conducted at two effector-to-target ratios (E:T): 5:1 and 10:1. Each point represents the average of three triplicate results. Granzyme B and CD107a levels were measured using fluorescent antibodies and detected by flow cytometry.

Supplementary Figure 3 Comparison of time to initial polarization of granules between NK cells from healthy donors and PHWC-19. Time to initial polarization was defined as the time between the start of a sustained contact between the NK cell and the tumor cell and the initial movement of the granules to the synapse. N = 10 conjugates for healthy and PHCW-19. Statistics were calculated using an unpaired T-test after testing for a normal distribution. P<0.05.

Supplementary Figure 4 Mass cytometry profiling of NK receptor co-expression on specific CD56^bright^ NK cell populations. **(A)** Representative t-SNE dimensionality reduction plots of the clusters within the CD56^bright^ CD57- (left) and CD56^bright^ CD57+ (right) NK cell populations from healthy donors (N = 15) and PHWC-19 (N = 17). **(B)** Pooled relative percentages of each cluster within the CD56^bright^ CD57- (left) and CD56^bright^ CD57+ (right) NK cell populations from healthy donors (N = 15) and PHWC-19 (N = 17) detected by single-cell mass cytometry. The legend applies to sections A and B. **(C)** Heatmap and dendrogram of log_2_ expression of activating and inhibitory receptors within the clusters of CD56^bright^ NK cell subset from whole blood of healthy donors (N = 15) and PHWC-19 (N = 17). Data was collected by single-cell mass cytometry. Lighter colors (larger numbers) on the legend indicate greater receptor expression than the darker colors (smaller numbers). Hierarchical clustering heat map was drawn using ChiPlot (https://www.chiplot.online/). * = P<0.05, ** = P<0.01, *** = P<0.001. Statistics for B were calculated using the Mann-Whitney test.

Supplementary Figure 5 Principal component analysis (PCA) of receptor expression in NK cell subsets. **(A)** Comparison of the receptor expression correlations in CD56^dim^ CD57- NK cells in healthy donors and PHWC-19. **(B)** Comparison of the receptor expression correlations in CD56^dim^ CD57+ NK cells in healthy donors and PHWC-19. **(C)** Comparison of the receptor expression correlations in CD56^bright^ CD57- NK cells in healthy donors and PHWC-19. **(D)** Comparison of the receptor expression correlations in CD56^bright^ CD57+ NK cells in healthy donors and PHWC-19. The NK cells were collected from the whole blood of healthy donors (N = 15) and PHWC-19 (N = 17). Data was collected by single-cell mass cytometry.

Supplementary Figure 6 Vaccine status and NK cell cytotoxicity in PHWC-19. Comparison of 721.221 tumor cell killing by NK cells from PHWC-19 that were known to be vaccinated to those that were not (N_Vax_=6–7 and N_No Vax_=4-5). Experiments were conducted at two effector-to-target ratios (E:T): 5:1 and 10:1.

Supplementary Figure 7 Comparisons of sex-related differences in granule-mediated mechanisms. **(A)** Paired comparison of the expression of intracellular effector molecules within CD56+CD3- PBMCs from healthy individuals and patients with COVID-19 for differences between sexes. The levels of intracellular effector molecules like granzymes A (N = 4), B (N = 10), K (N = 4), perforin (N = 9), and granulysin (N = 10) were measured using fluorescent antibodies and detected by flow cytometry. **(B)** Comparison of the degranulation levels of intracellular granules from NK cells from healthy individuals and patients with COVID-19 for differences between sexes. The levels of CD107a were tagged using fluorescent antibodies (anti-CD107a) after the NK cells were co-cultured with 721.221 tumor cells and detected by flow cytometry. N = 6. Experiments were conducted at two effector-to-target ratios (E:T): 5:1 and 10:1. **(C)** Comparison of the level of extracellular effector molecules from NK cells from healthy individuals and patients with COVID-19 for differences between sexes. The granzyme A (N = 6), granzyme B (N = 10), and perforin (N = 4) levels in the supernatants collected from a co-culture of the pNKs with 721.221 tumor cells were detected using ELISA. Experiments were conducted at one effector-to-target ratio (E:T) of 10:1. Males are on the left side, while females are on the right side of each panel. Circles indicate healthy donors, while triangles indicate patients hospitalized with COVID-19. * = P<0.05, ** = P<0.01, *** = P<0.001; calculated using the Kruskal-Wallis ANOVA with Dunn’s multiple comparisons test.

Supplementary Figure 8 NK cell cytotoxicity defects were less pronounced in male PHWC-19 treated with dexamethasone than in male PHWC-19 not treated with dexamethasone. Circles indicate healthy donors, and triangles indicate patients hospitalized with COVID-19. To determine the effect of dexamethasone on NK cells from healthy subjects, dexamethasone was added to the cultured media (0.1 μM) (N_Healthy_=24 and N_COVID_=14). Experiments were conducted at two effector-to-target ratios (E:T): 5:1 and 10:1. Each point represents a triplicate result’s average for each subject. Statistics were calculated using the Mann-Whitney test; * = P < 0.05.

Supplementary Table 1 Flow cytometry antibody panel.

Supplementary Table 2 CYTOF antibody panel.
