# Supplementary figures and images for: The effect of COVID-19 and sex differences on natural killer cell cytotoxicity
Source: Front Cell Infect Microbiol. 2025 Sep 22;15:1635043. doi: 10.3389/fcimb.2025.1635043 (PMC12497742; doi:10.3389/fcimb.2025.1635043)

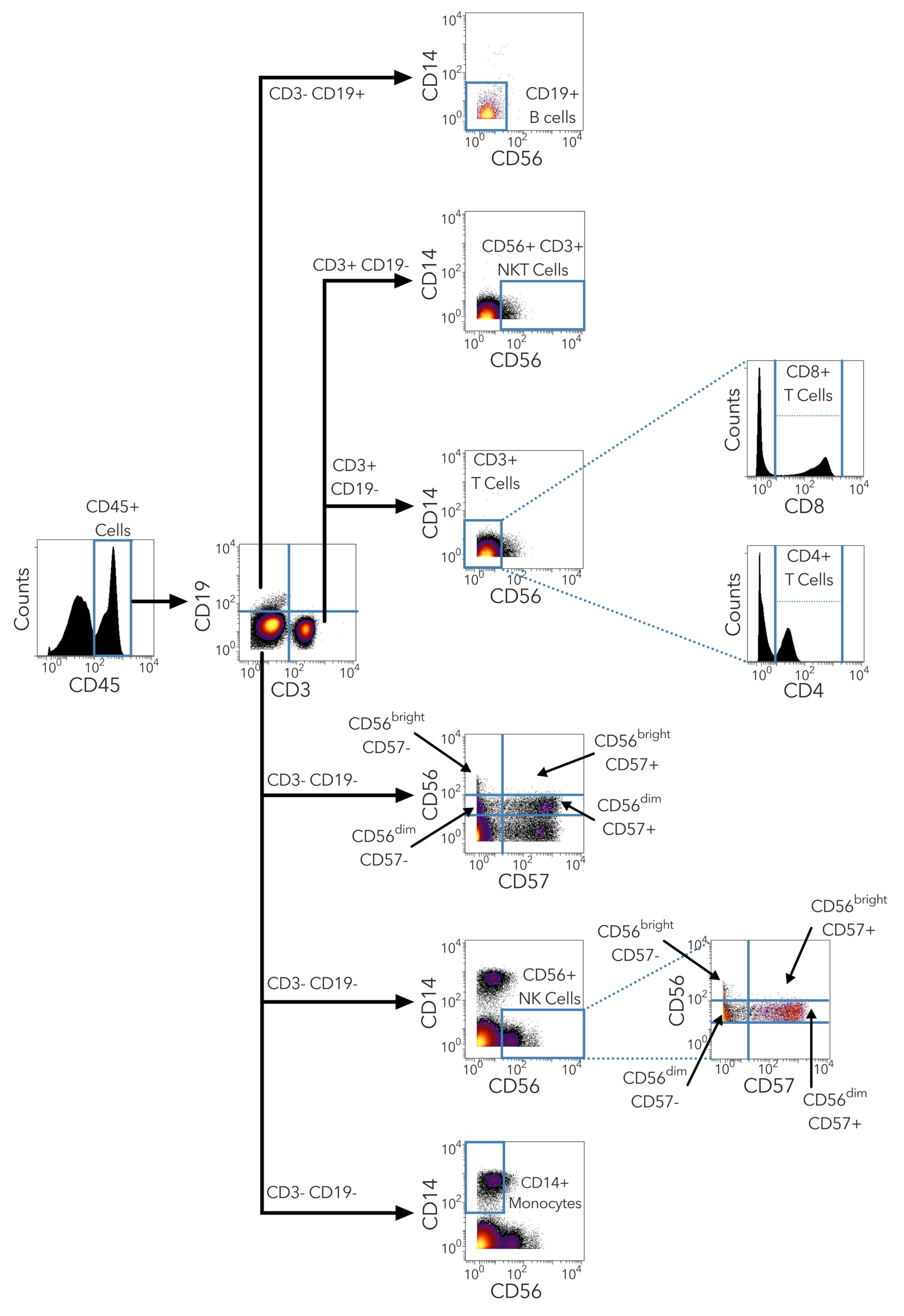

Supplement: Supplementary Figure 1 — Gating strategy for mass cytometry. The HELIOS machine was used for acquisition, and the data was uploaded to the Cytobank program, where manual hierarchical gating was performed after barcoding and labelling with lanthanide–labelled antibodies. All cell populations were initially gated on CD45+ to identify white blood cells via a histogram. Cellular populations were further identified with the following definitions: B cells (CD45+ CD19+), NKT Cells (CD45+ CD3+ CD56+), CD8+ T cells (CD45+ CD3+ CD8+), CD4+ T cells (CD45+ CD3+ CD4+), NK cells (CD45+ NKp46+), and monocytes (CD45+ CD14+). NK cells were further stratified into subsets of CD56dim CD57-, CD56dim CD57+, CD56bright CD57-, and CD56bright CD57 +. No signal was detected below zero, which caused the abrupt cutoff. [file Image1.tif]

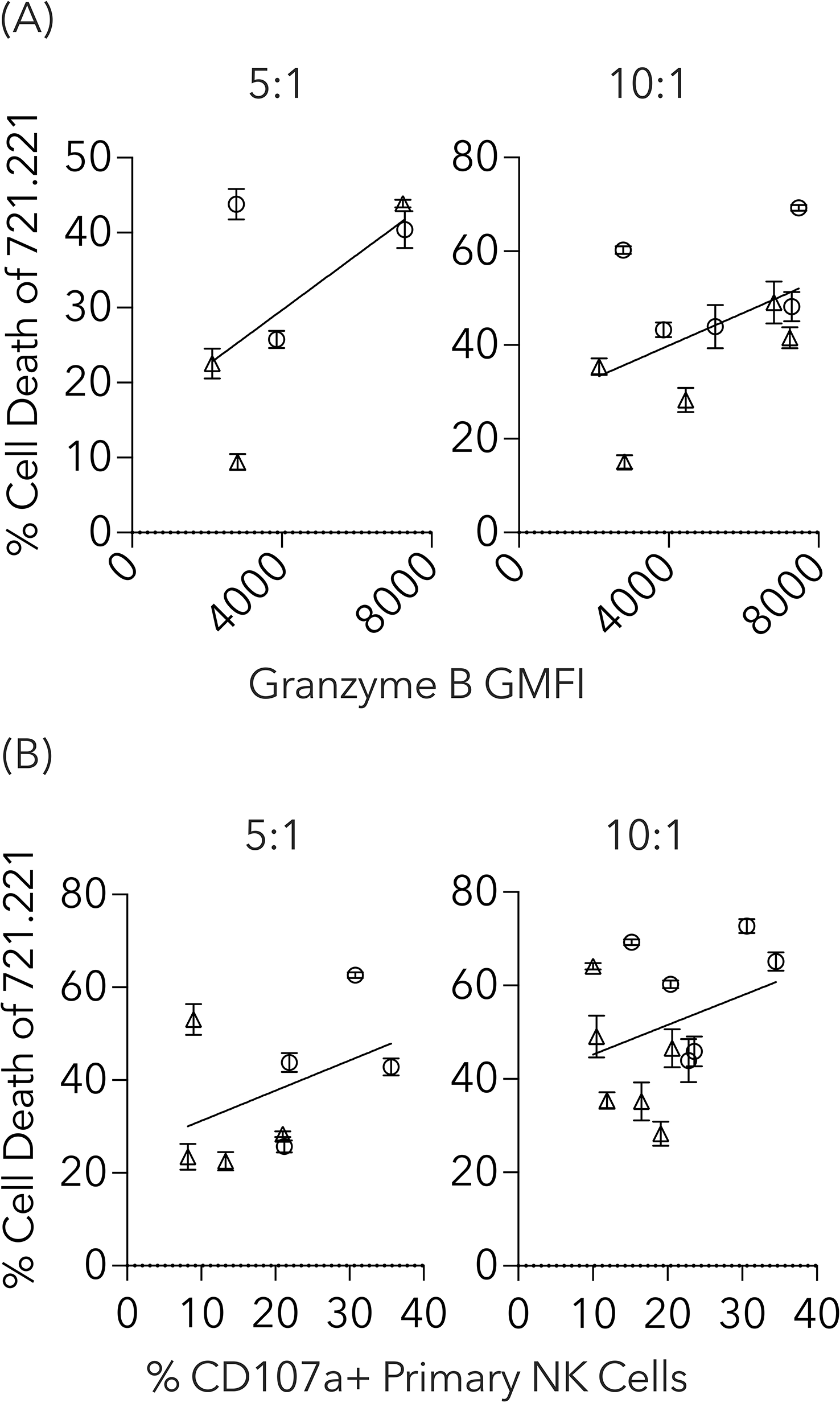

Supplement: Supplementary Figure 2 — The correlation of granzyme B expression and degranulation with cytotoxicity. (A) 721.221 tumor cell killing by NK cells increases with increasing intracellular granzyme B levels in NK cells from healthy individuals and PHWC-19 (NHealthy=8 and NCOVID=8) (B) 721.221 tumor cell killing by NK cells increases with increasing degranulation in NK cells from healthy individuals and PHWC-19 (NHealthy=10 and NCOVID=10). Circles indicate healthy individuals, while triangles indicate PHWC-19. Experiments were conducted at two effector-to-target ratios (E:T): 5:1 and 10:1. Each point represents the average of three triplicate results. Granzyme B and CD107a levels were measured using fluorescent antibodies and detected by flow cytometry. [file Image2.tif]

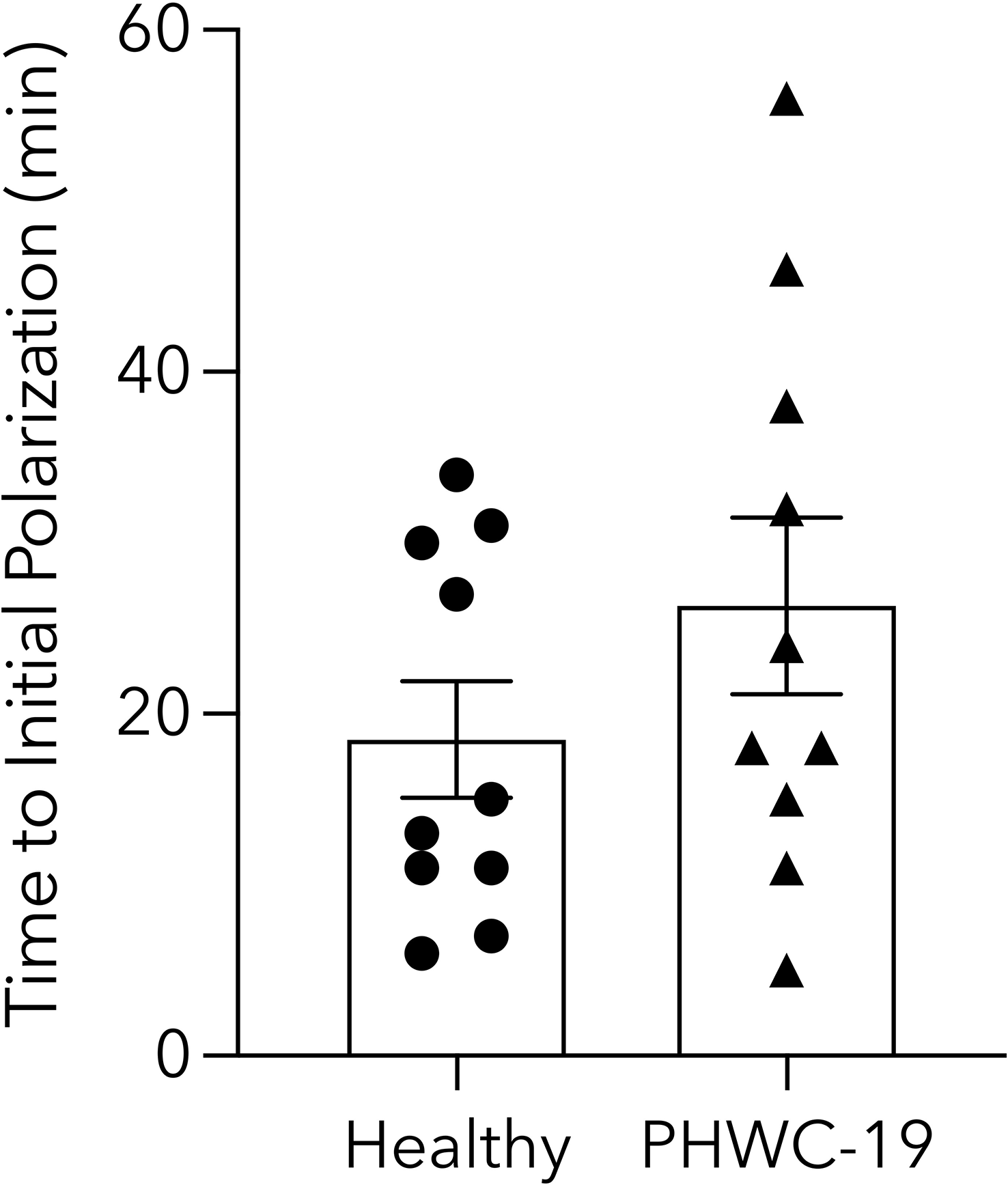

Supplement: Supplementary Figure 3 — Comparison of time to initial polarization of granules between NK cells from healthy donors and PHWC-19. Time to initial polarization was defined as the time between the start of a sustained contact between the NK cell and the tumor cell and the initial movement of the granules to the synapse. N = 10 conjugates for healthy and PHCW-19. Statistics were calculated using an unpaired T-test after testing for a normal distribution. P<0.05. [file Image3.tif]

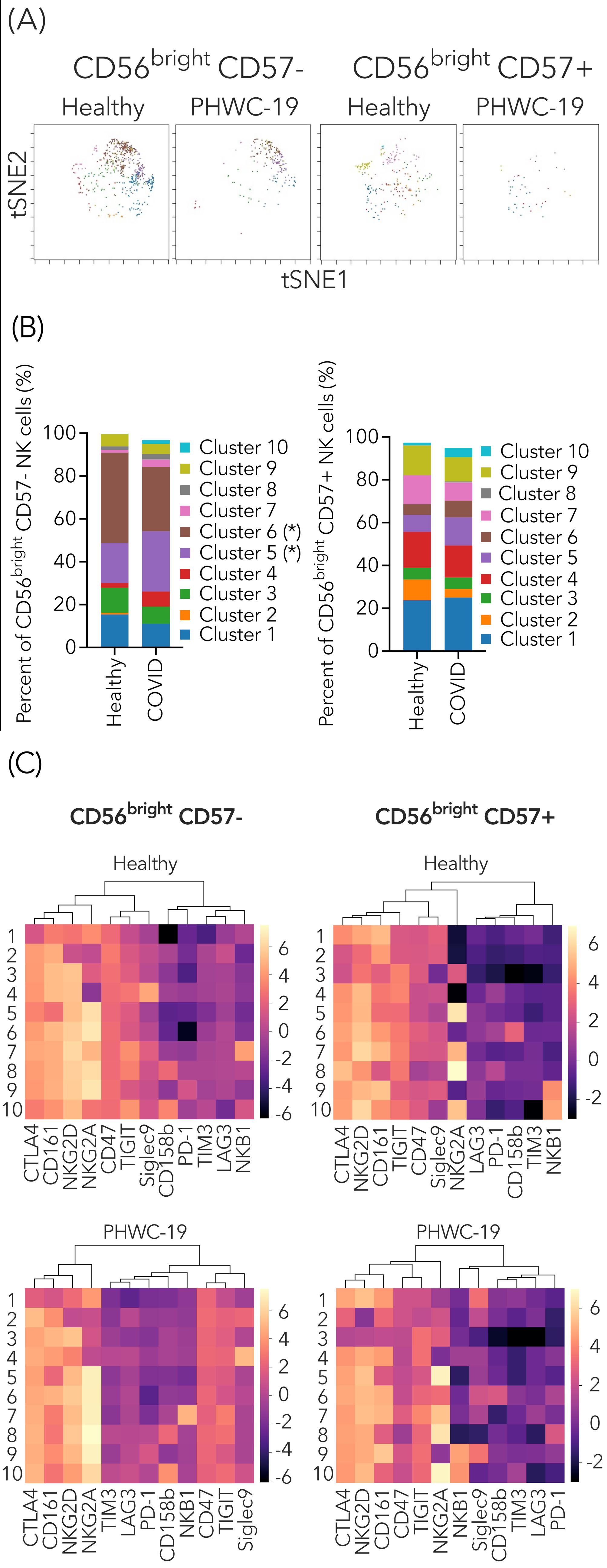

Supplement: Supplementary Figure 4 — Mass cytometry profiling of NK receptor co-expression on specific CD56bright NK cell populations. (A) Representative t-SNE dimensionality reduction plots of the clusters within the CD56bright CD57- (left) and CD56bright CD57+ (right) NK cell populations from healthy donors (N = 15) and PHWC-19 (N = 17). (B) Pooled relative percentages of each cluster within the CD56bright CD57- (left) and CD56bright CD57+ (right) NK cell populations from healthy donors (N = 15) and PHWC-19 (N = 17) detected by single-cell mass cytometry. The legend applies to sections A and B. (C) Heatmap and dendrogram of log2 expression of activating and inhibitory receptors within the clusters of CD56bright NK cell subset from whole blood of healthy donors (N = 15) and PHWC-19 (N = 17). Data was collected by single-cell mass cytometry. Lighter colors (larger numbers) on the legend indicate greater receptor expression than the darker colors (smaller numbers). Hierarchical clustering heat map was drawn using ChiPlot (https://www.chiplot.online/). * = P<0.05, ** = P<0.01, *** = P<0.001. Statistics for B were calculated using the Mann-Whitney test. [file Image4.tif]

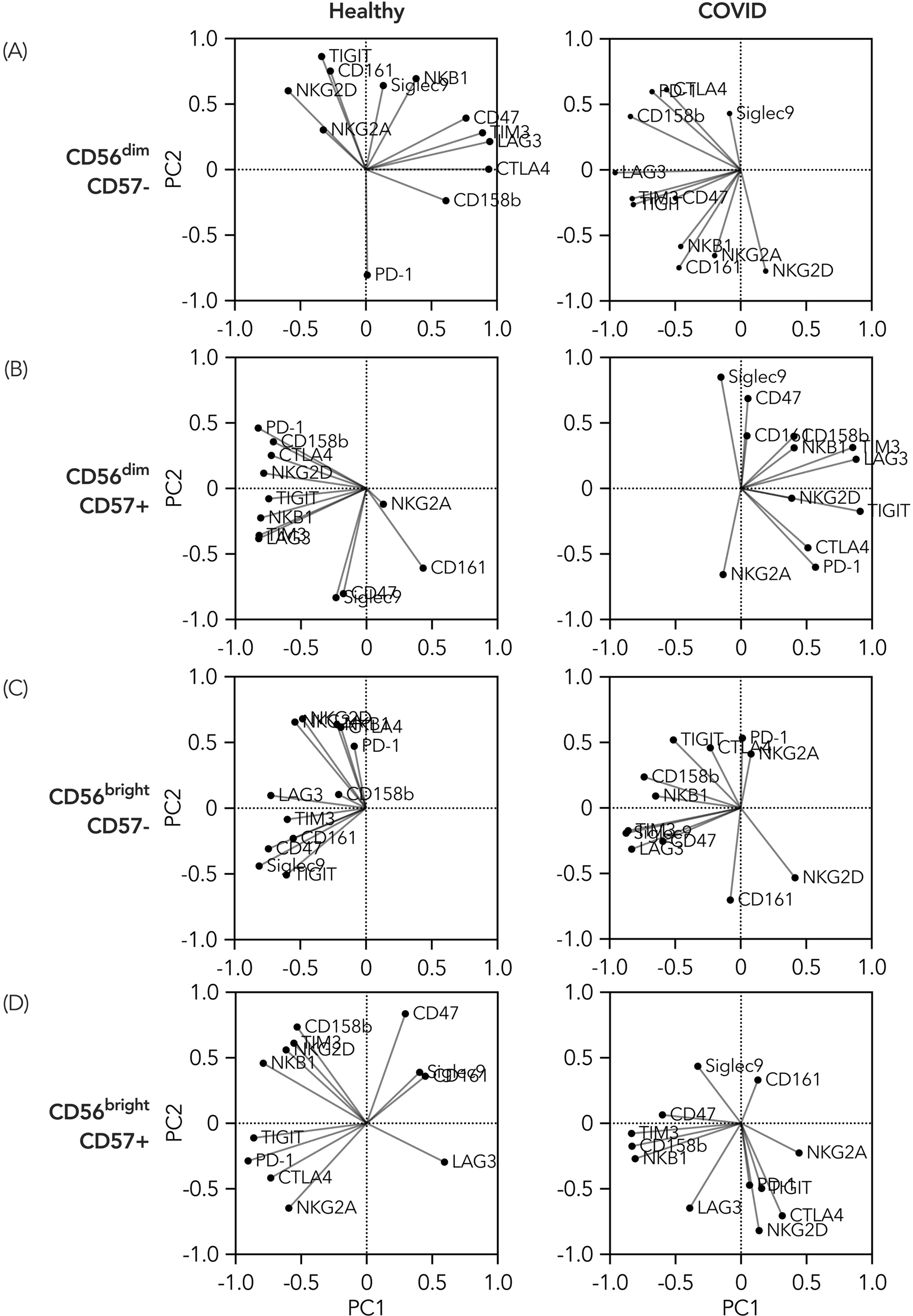

Supplement: Supplementary Figure 5 — Principal component analysis (PCA) of receptor expression in NK cell subsets. (A) Comparison of the receptor expression correlations in CD56dim CD57- NK cells in healthy donors and PHWC-19. (B) Comparison of the receptor expression correlations in CD56dim CD57+ NK cells in healthy donors and PHWC-19. (C) Comparison of the receptor expression correlations in CD56bright CD57- NK cells in healthy donors and PHWC-19. (D) Comparison of the receptor expression correlations in CD56bright CD57+ NK cells in healthy donors and PHWC-19. The NK cells were collected from the whole blood of healthy donors (N = 15) and PHWC-19 (N = 17). Data was collected by single-cell mass cytometry. [file Image5.tif]

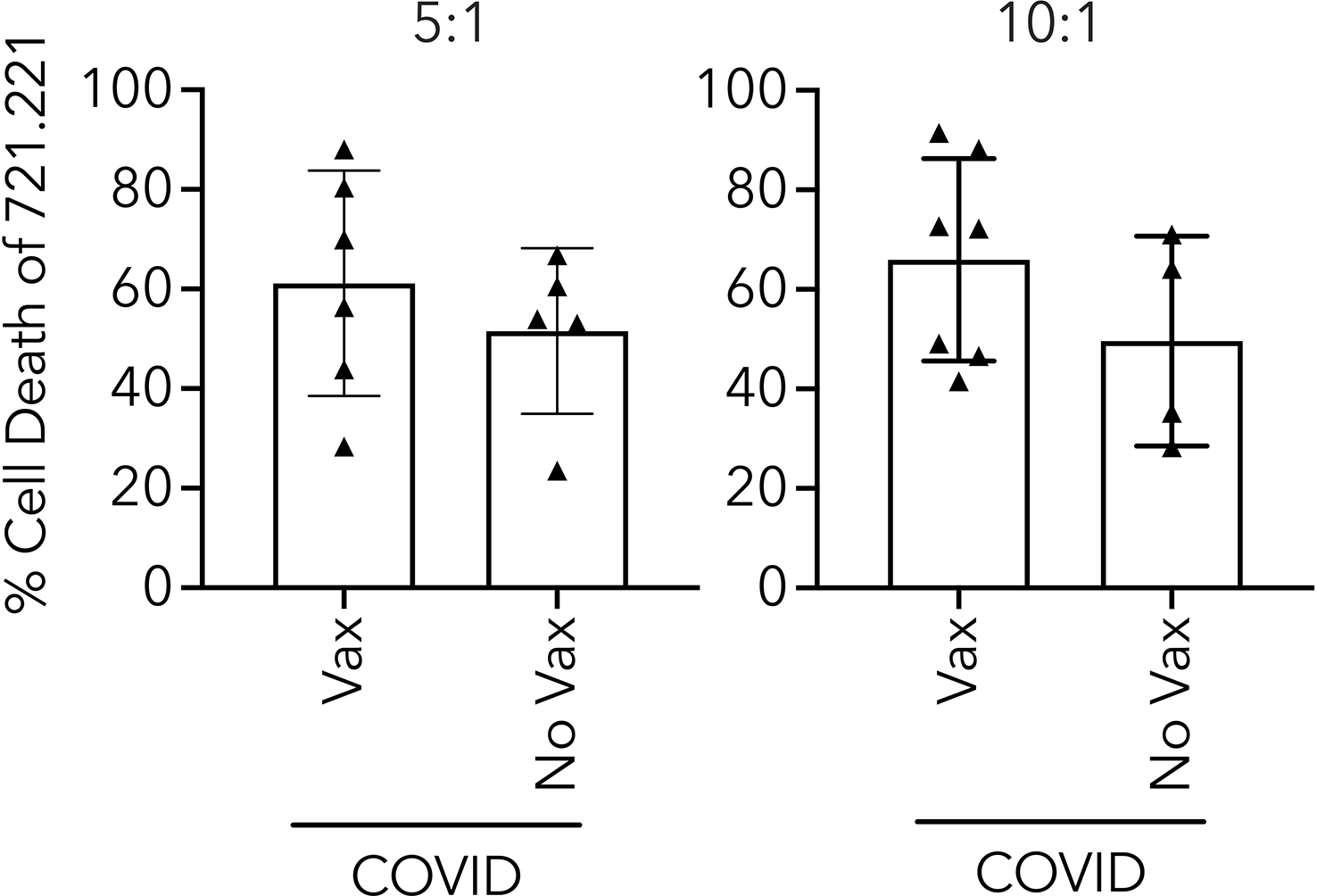

Supplement: Supplementary Figure 6 — Vaccine status and NK cell cytotoxicity in PHWC-19. Comparison of 721.221 tumor cell killing by NK cells from PHWC-19 that were known to be vaccinated to those that were not (NVax=6–7 and NNo Vax=4-5). Experiments were conducted at two effector-to-target ratios (E:T): 5:1 and 10:1. [file Image6.tif]

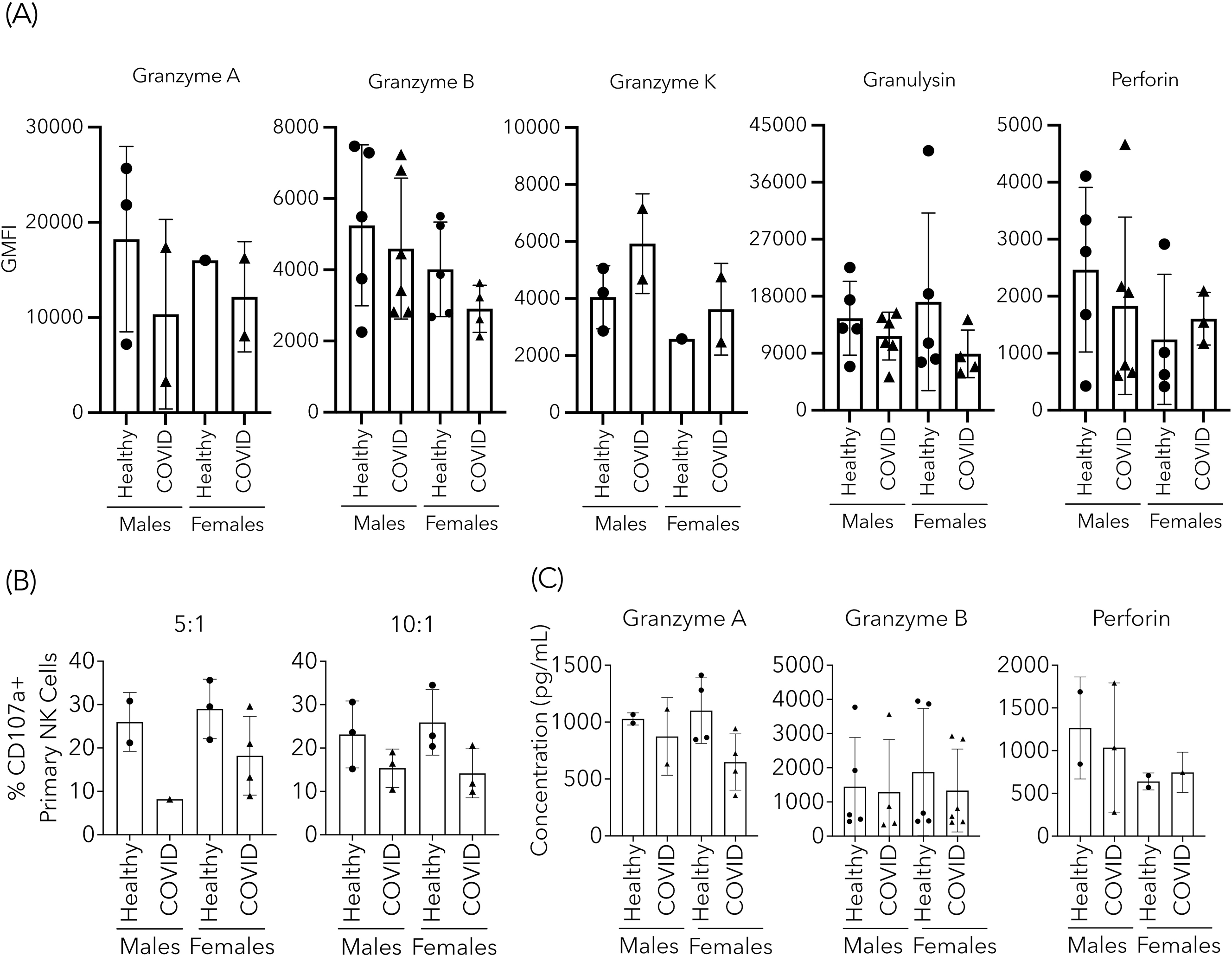

Supplement: Supplementary Figure 7 — Comparisons of sex-related differences in granule-mediated mechanisms. (A) Paired comparison of the expression of intracellular effector molecules within CD56+CD3- PBMCs from healthy individuals and patients with COVID-19 for differences between sexes. The levels of intracellular effector molecules like granzymes A (N = 4), B (N = 10), K (N = 4), perforin (N = 9), and granulysin (N = 10) were measured using fluorescent antibodies and detected by flow cytometry. (B) Comparison of the degranulation levels of intracellular granules from NK cells from healthy individuals and patients with COVID-19 for differences between sexes. The levels of CD107a were tagged using fluorescent antibodies (anti-CD107a) after the NK cells were co-cultured with 721.221 tumor cells and detected by flow cytometry. N = 6. Experiments were conducted at two effector-to-target ratios (E:T): 5:1 and 10:1. (C) Comparison of the level of extracellular effector molecules from NK cells from healthy individuals and patients with COVID-19 for differences between sexes. The granzyme A (N = 6), granzyme B (N = 10), and perforin (N = 4) levels in the supernatants collected from a co-culture of the pNKs with 721.221 tumor cells were detected using ELISA. Experiments were conducted at one effector-to-target ratio (E:T) of 10:1. Males are on the left side, while females are on the right side of each panel. Circles indicate healthy donors, while triangles indicate patients hospitalized with COVID-19. * = P<0.05, ** = P<0.01, *** = P<0.001; calculated using the Kruskal-Wallis ANOVA with Dunn’s multiple comparisons test. [file Image7.tif]

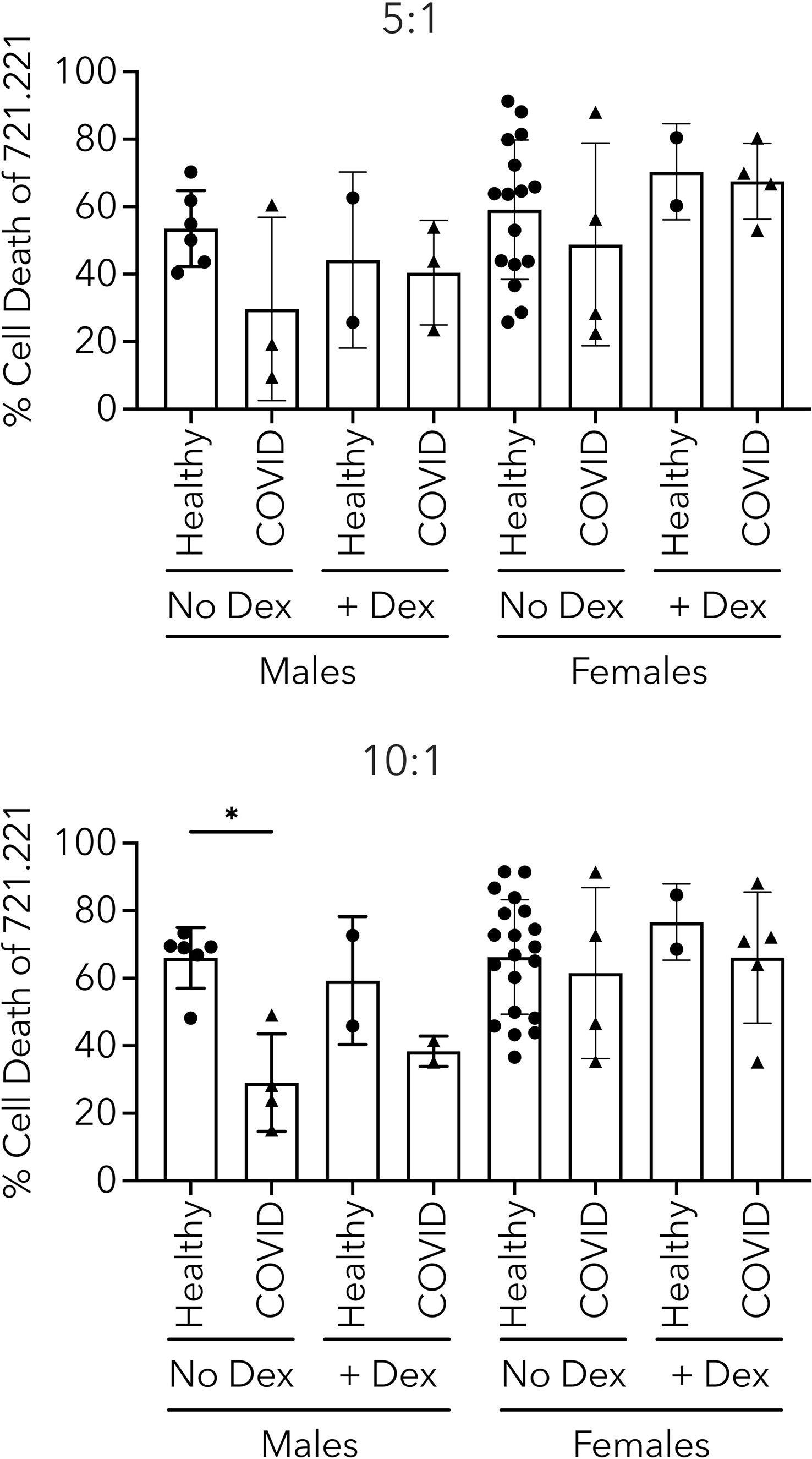

Supplement: Supplementary Figure 8 — NK cell cytotoxicity defects were less pronounced in male PHWC-19 treated with dexamethasone than in male PHWC-19 not treated with dexamethasone. Circles indicate healthy donors, and triangles indicate patients hospitalized with COVID-19. To determine the effect of dexamethasone on NK cells from healthy subjects, dexamethasone was added to the cultured media (0.1 μM) (NHealthy=24 and NCOVID=14). Experiments were conducted at two effector-to-target ratios (E:T): 5:1 and 10:1. Each point represents a triplicate result’s average for each subject. Statistics were calculated using the Mann-Whitney test; * = P < 0.05. [file Image8.tif]

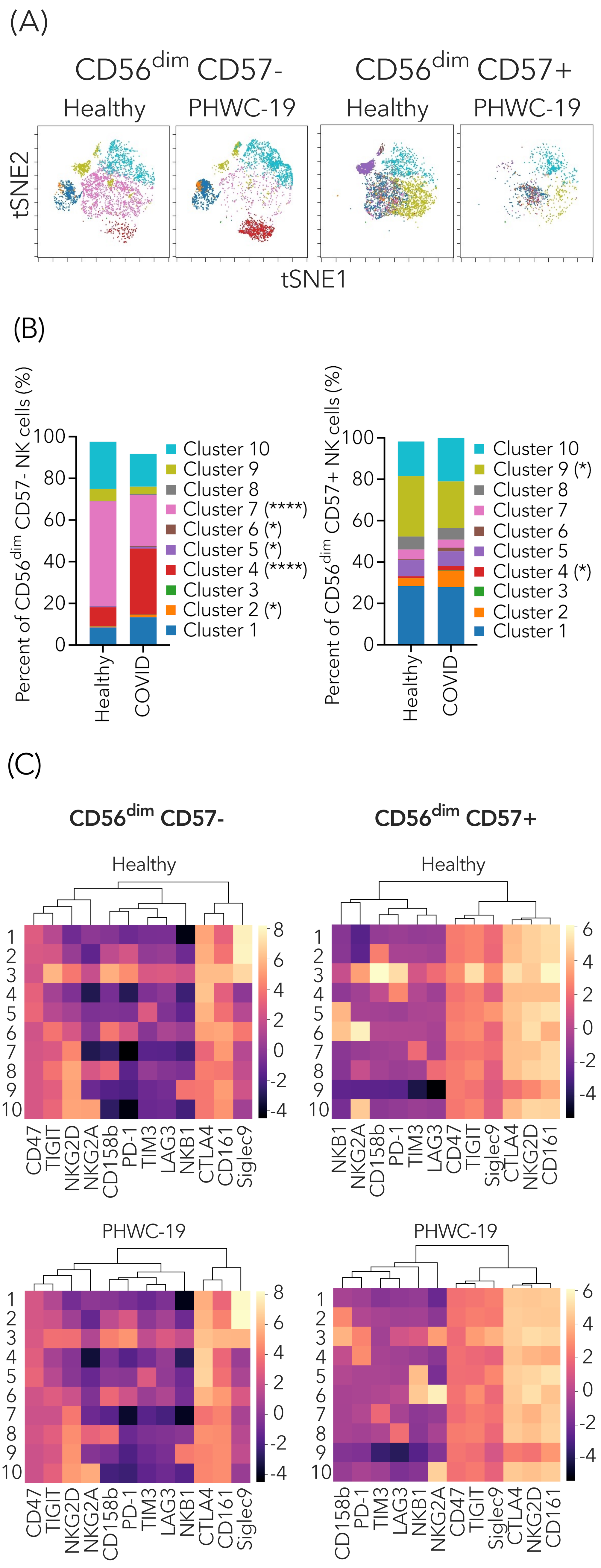

Supplement: Supplementary file 13 [file DataSheet1.zip › Paper Figures/Figure 6.tif]

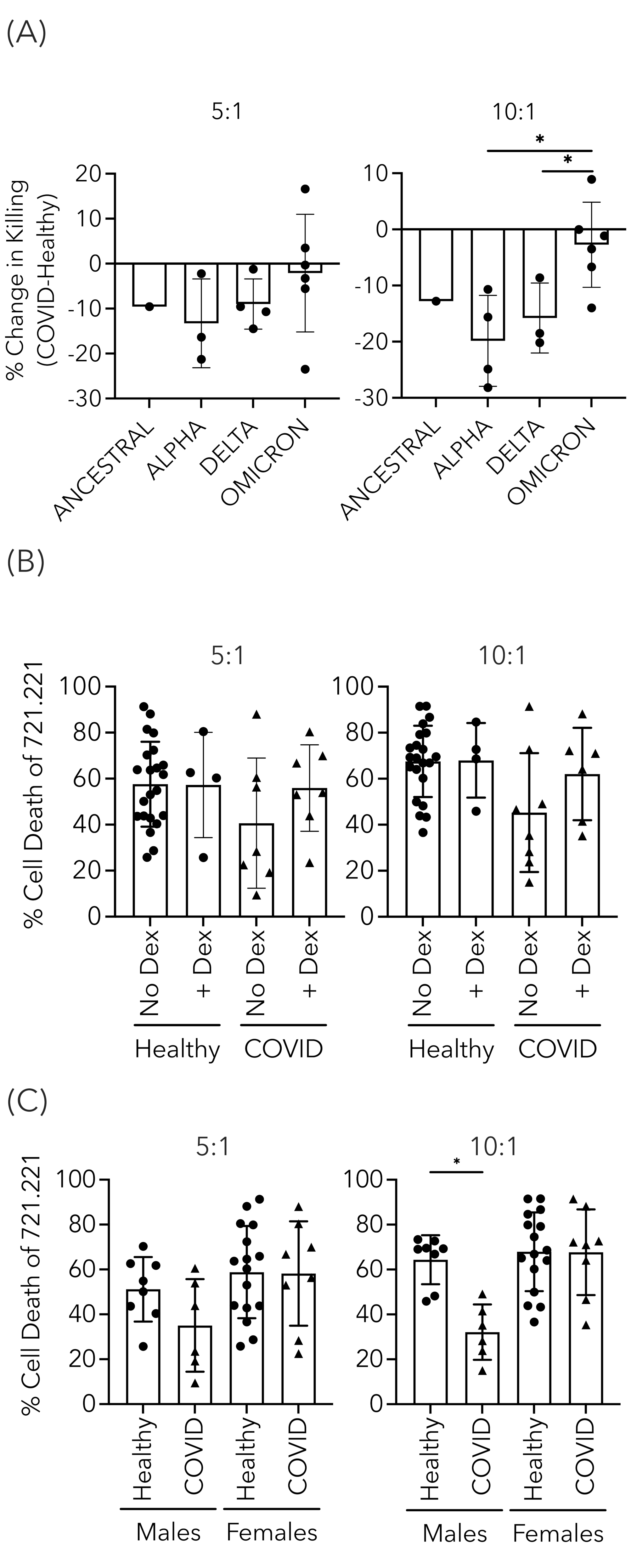

Supplement: Supplementary file 13 [file DataSheet1.zip › Paper Figures/Figure 7.tif]

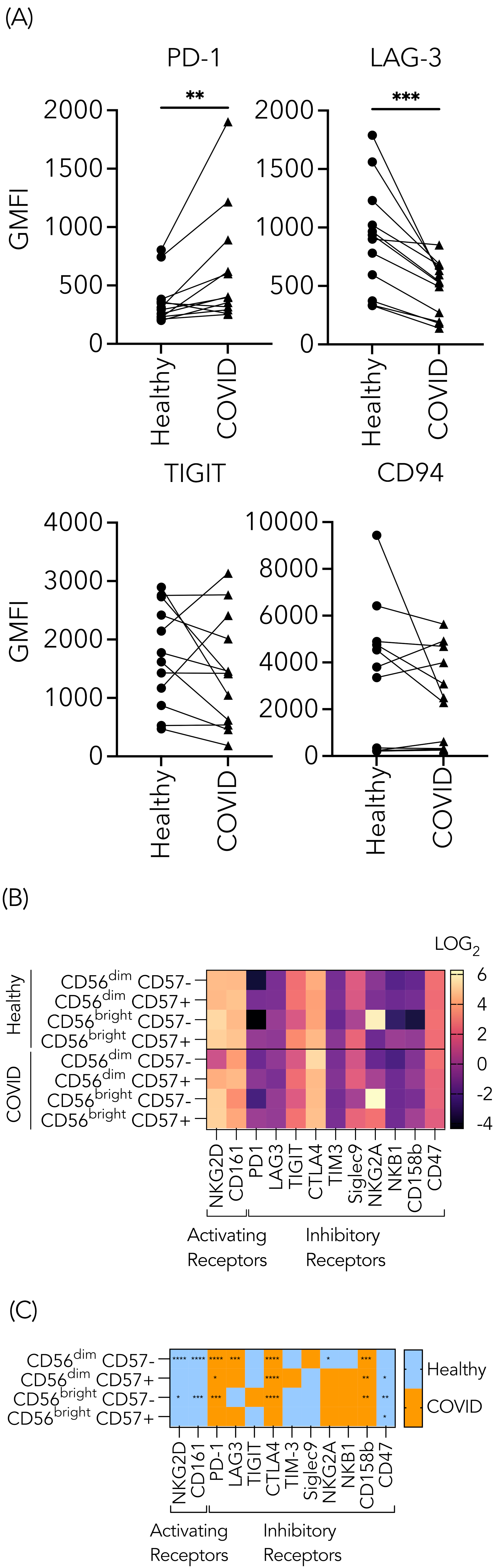

Supplement: Supplementary file 13 [file DataSheet1.zip › Paper Figures/Figure 5.tif]

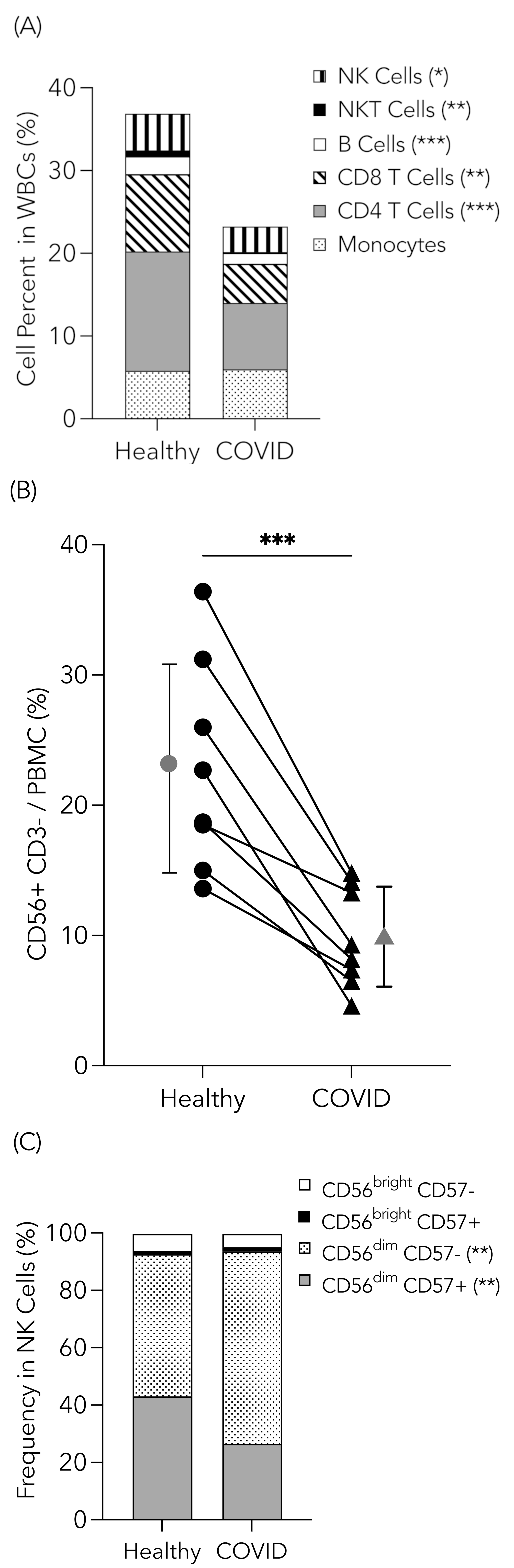

Supplement: Supplementary file 13 [file DataSheet1.zip › Paper Figures/Figure 1.tif]

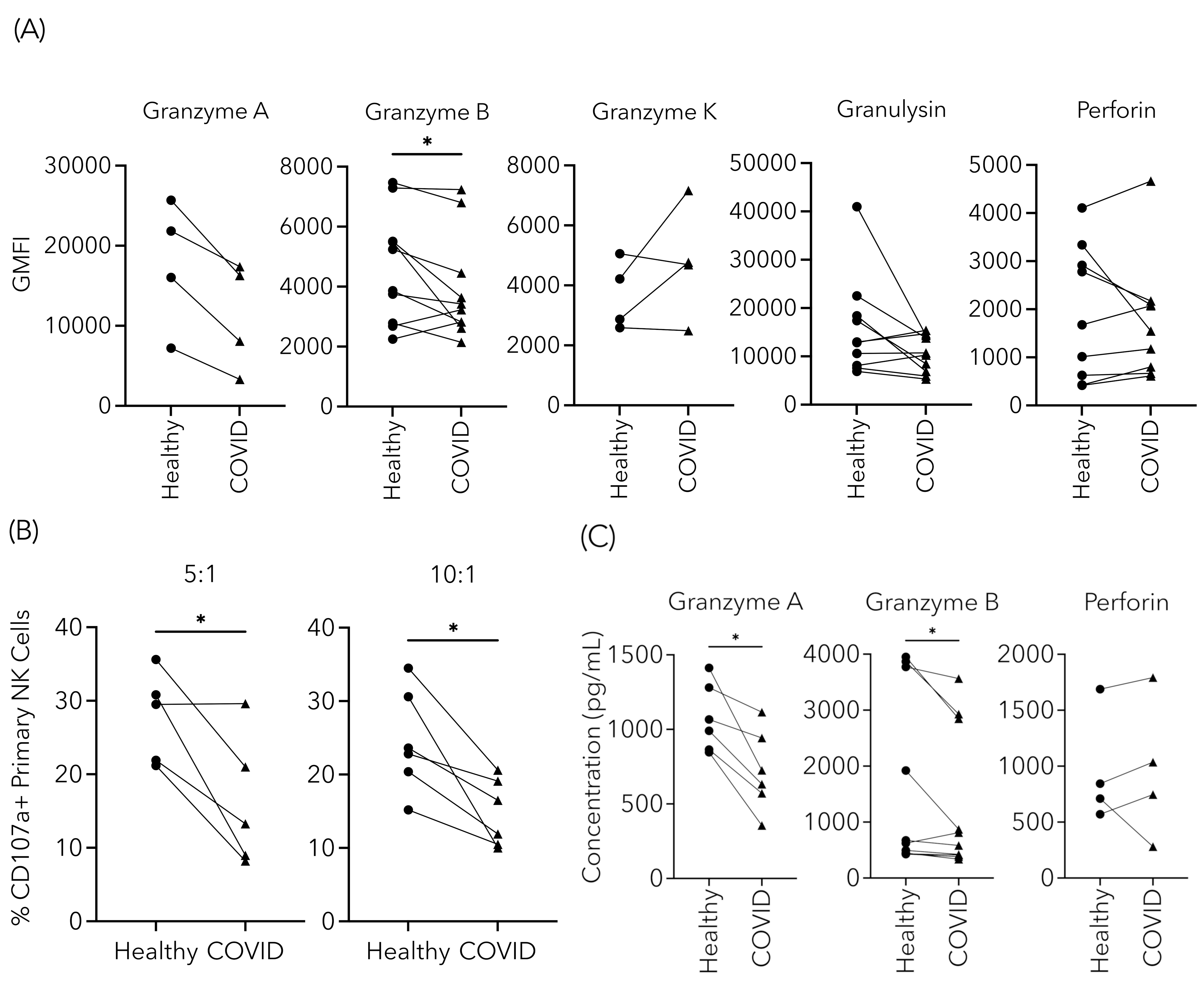

Supplement: Supplementary file 13 [file DataSheet1.zip › Paper Figures/Figure 3.tif]

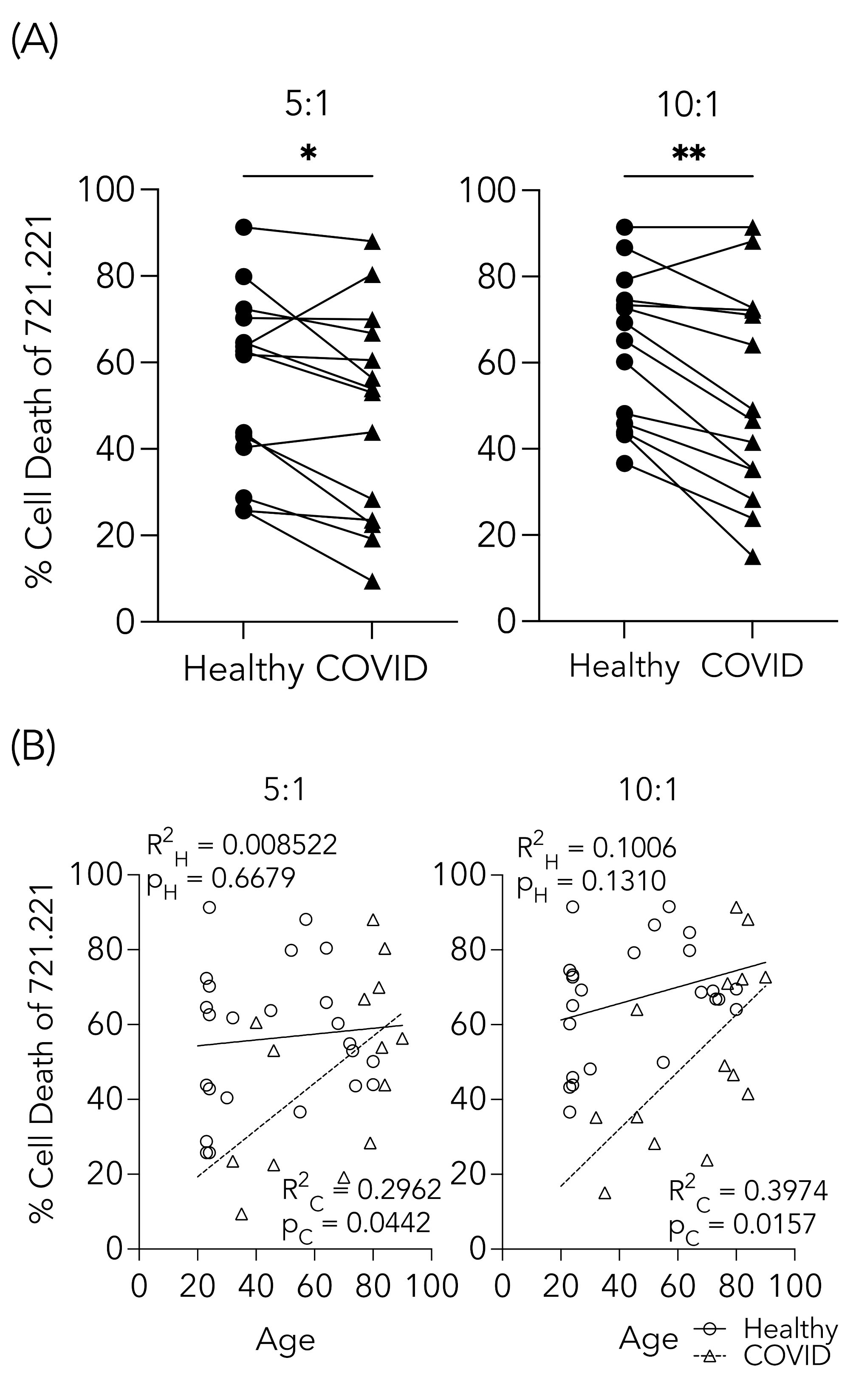

Supplement: Supplementary file 13 [file DataSheet1.zip › Paper Figures/Figure 2.tif]

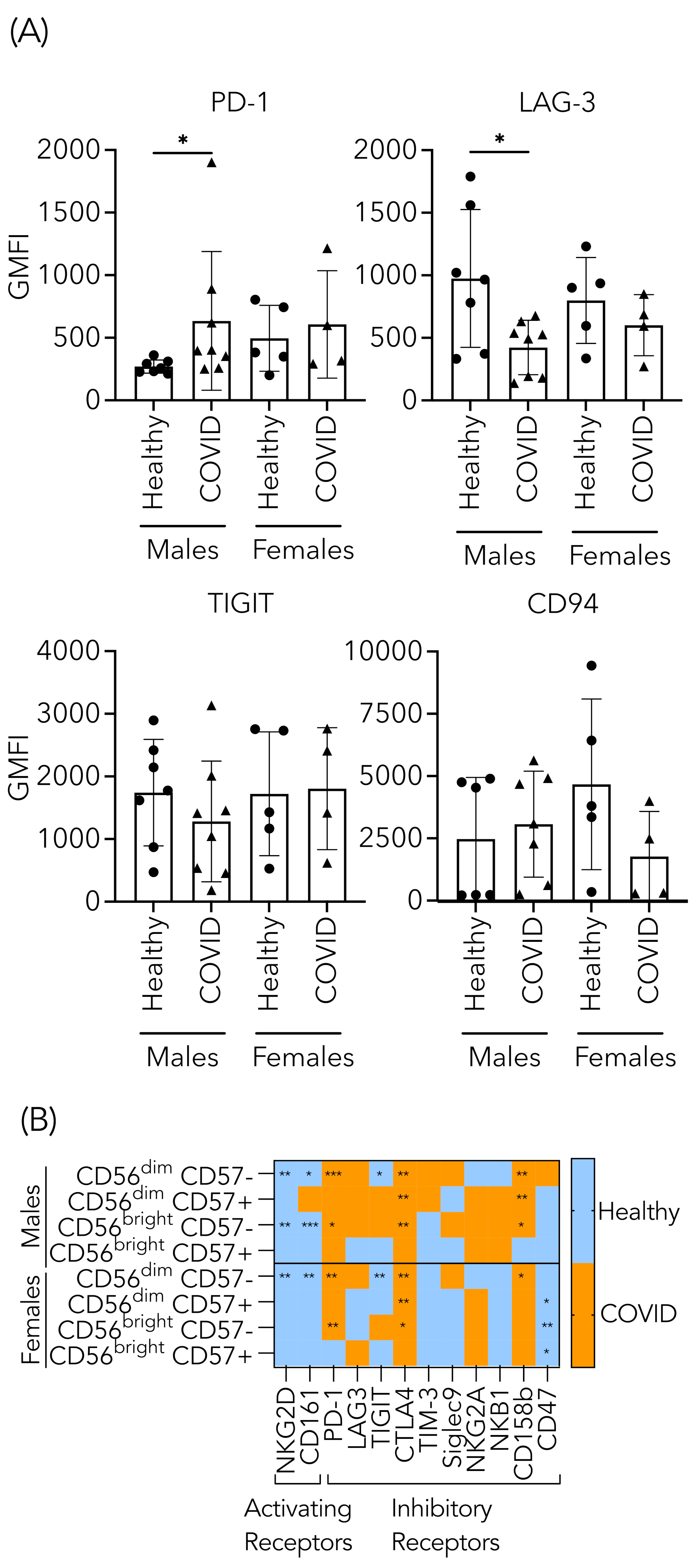

Supplement: Supplementary file 13 [file DataSheet1.zip › Paper Figures/Figure 8.tif]

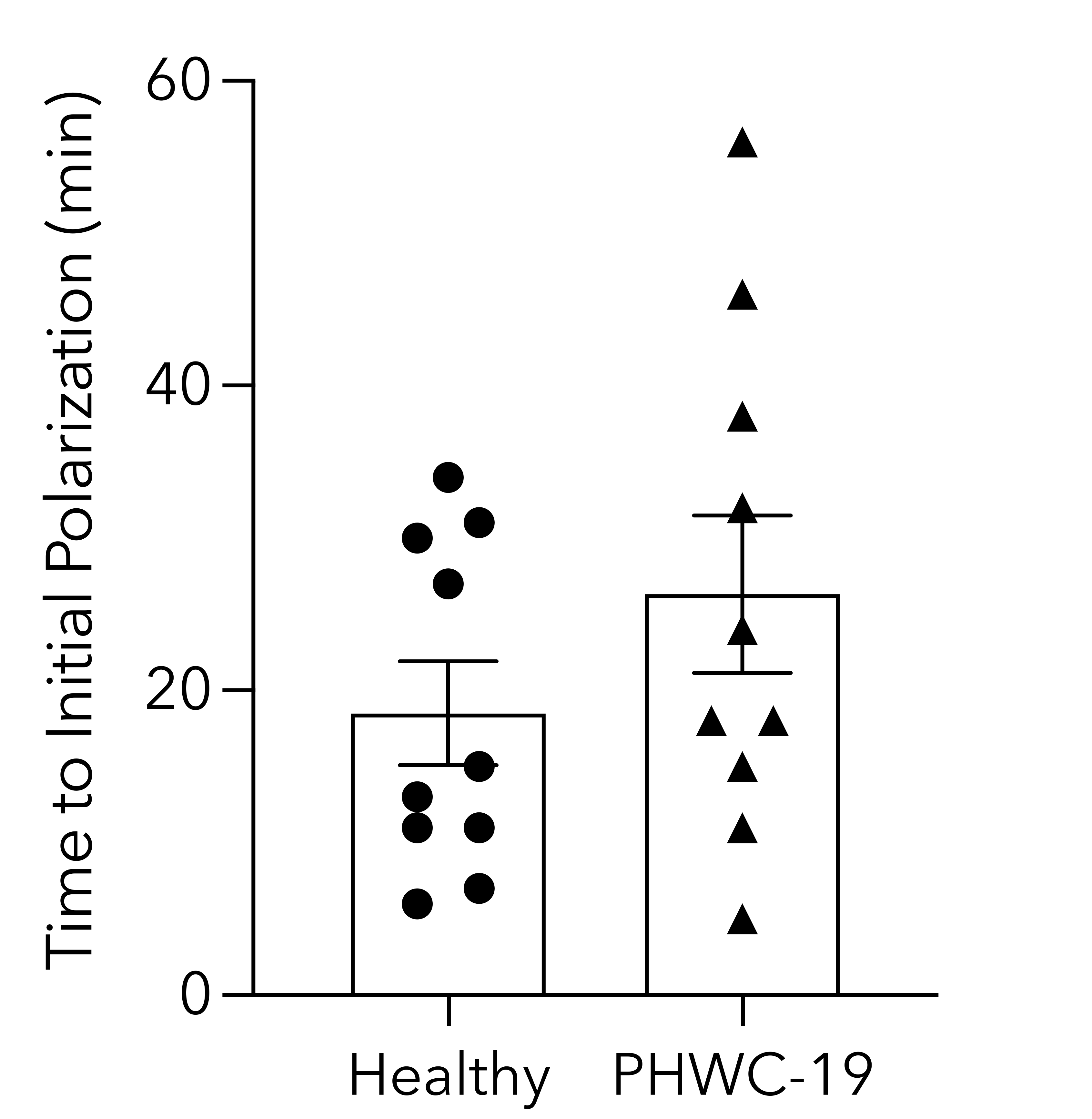

Supplement: Supplementary file 14 [file DataSheet2.zip › Supplementary Figures/Sup Figure 3.tif]

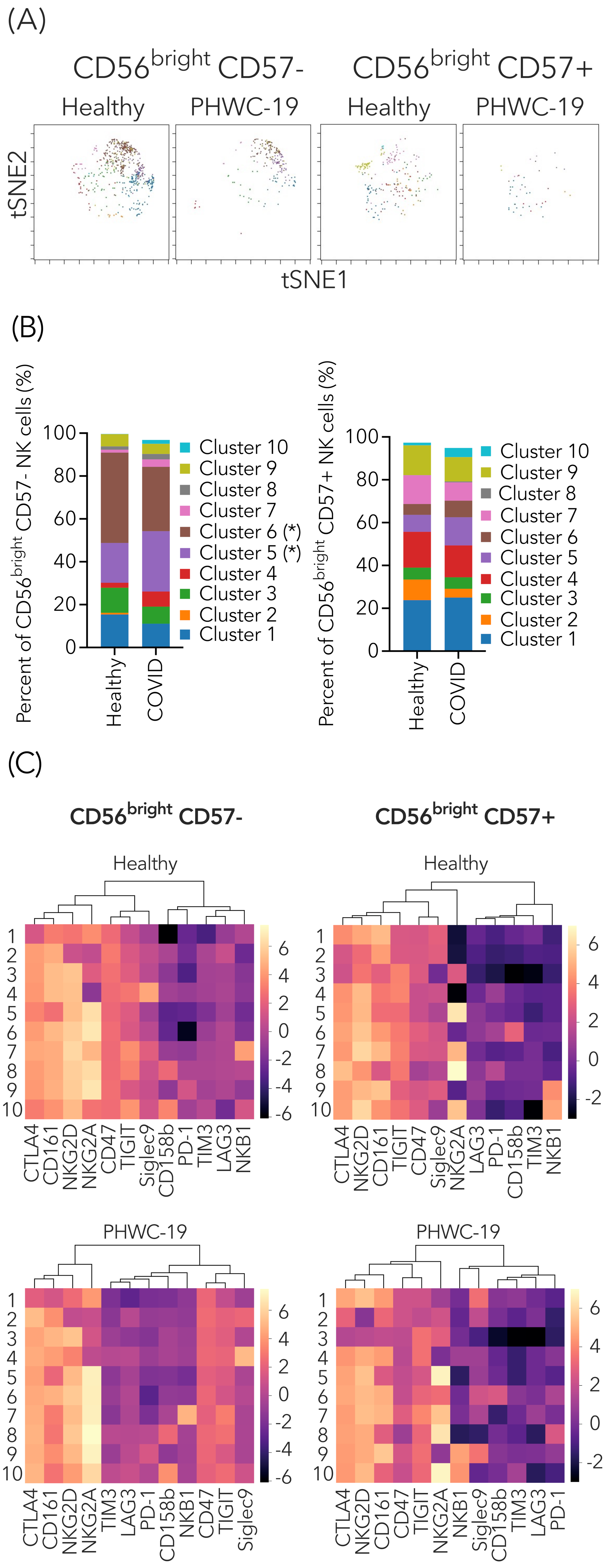

Supplement: Supplementary file 14 [file DataSheet2.zip › Supplementary Figures/Sup Figure 4.tif]

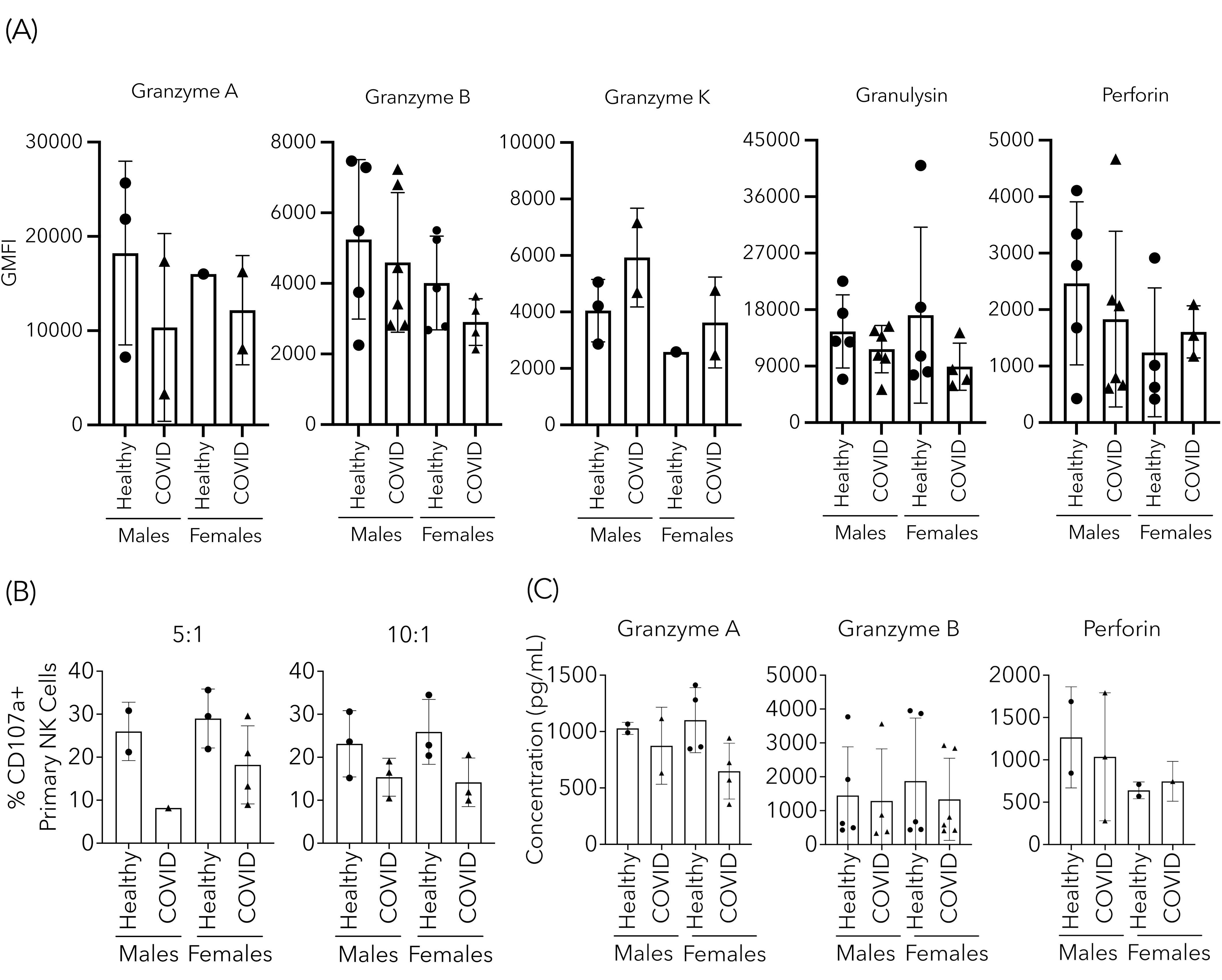

Supplement: Supplementary file 14 [file DataSheet2.zip › Supplementary Figures/Sup Figure 7.tif]

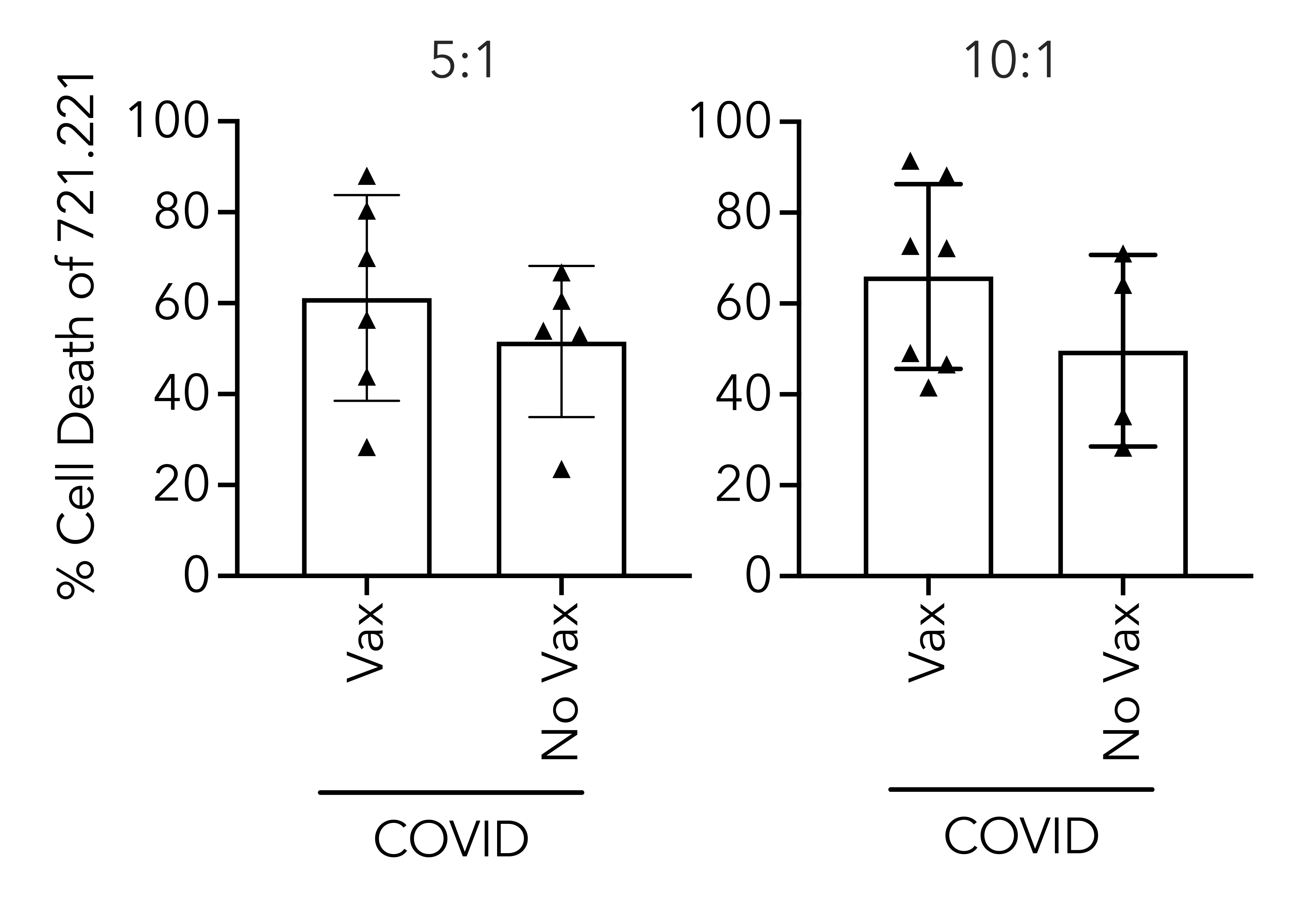

Supplement: Supplementary file 14 [file DataSheet2.zip › Supplementary Figures/Sup Figure 6.tif]

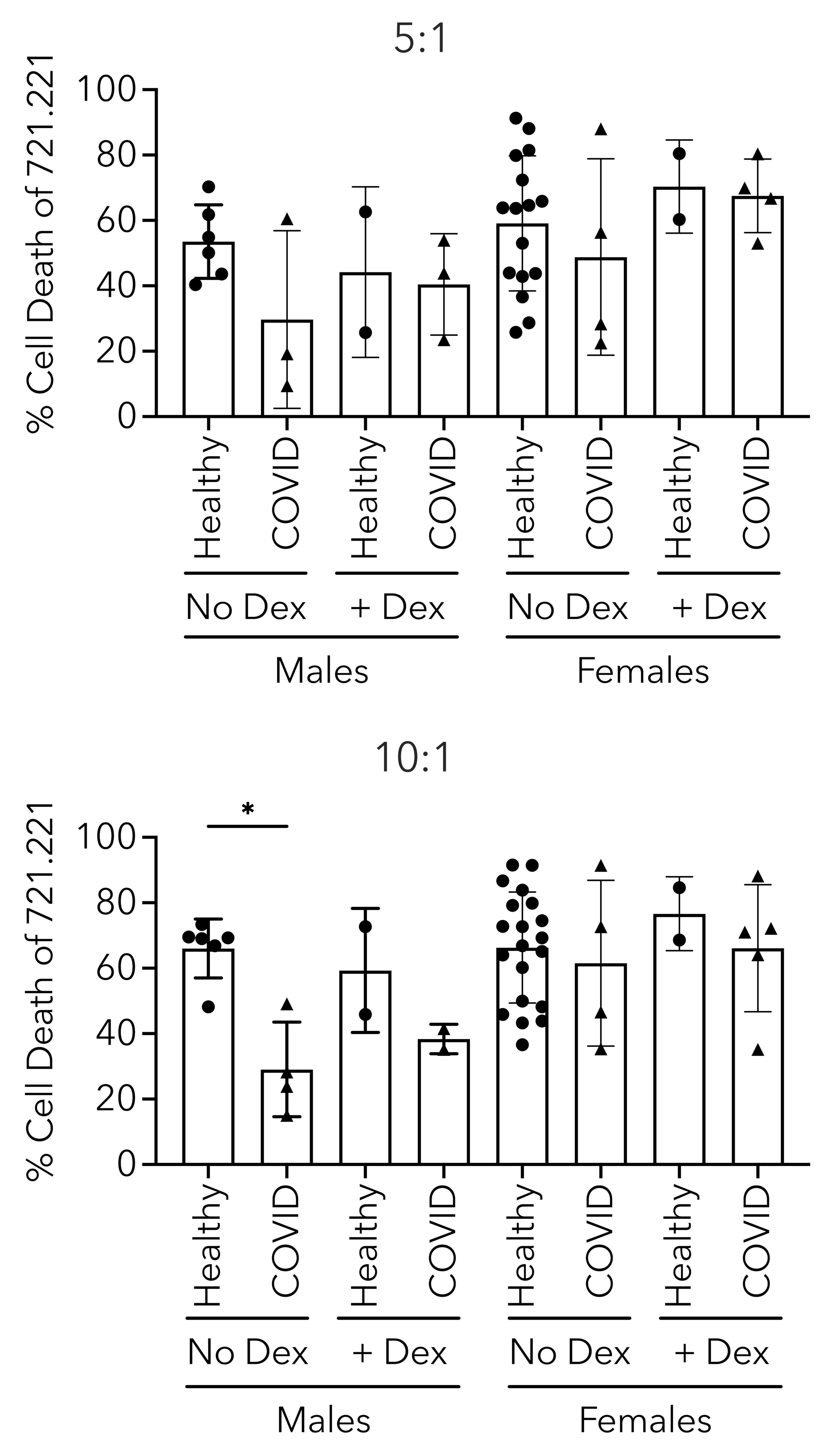

Supplement: Supplementary file 14 [file DataSheet2.zip › Supplementary Figures/Sup Figure 8.tif]
