# Supplementary material for: The effect of COVID-19 and sex differences on natural killer cell cytotoxicity
Source: Front Cell Infect Microbiol. 2025 Sep 22;15:1635043. doi: 10.3389/fcimb.2025.1635043 (PMC12497742; doi:10.3389/fcimb.2025.1635043)
Supplement: Supplementary Table 1 — Flow cytometry antibody panel. [file Table1.docx]

**Supplementary Table 1. Flow cytometry antibody panel.**

| **Marker** | **Clone** | **Fluorescent Dye** | **Source** | **Catalogue #** |
| --- | --- | --- | --- | --- |
| CD56 | HCD56 | Alexa Fluor 488 | Biolegend | 318312 |
| CD56 | HCD56 | PE | Biolegend | 318306 |
| CD3 | UCHT1 | Alexa Fluor 488 | Biolegend | 300415 |
| CD3 | SK7 | PerCP Cy5.5 | Invitrogen/eBioscience | 45-0036-42 |
| Granzyme A | REA162 | PE | Miltenyi Biotec | 130-123-973 |
| Granzyme B | GB11 | PE | Invitrogen/eBioscience | 12-8899-41 |
| Granzyme K | GM6C3 | PE | Santa Cruz | sc-56125 |
| Perforin | δG9 | PE | BD Pharmingen | 556437 |
| Granulysin | RB1 | Alexa Fluor 488 | BD Pharmingen | 558254 |
| PD-1 | MIH4 | PE | Invitrogen/eBioscience | 12-9969-41 |
| LAG-3 | 3DS223H | PE | Invitrogen/eBioscience | 12-2239-41 |
| TIGIT | MBSA43 | PE | Invitrogen/eBioscience | 12-9500-41 |
| CD94 | HP-3D9 | FITC | BD Pharmingen | 555888 |
| IgG1 Isotype | P3.6.2.8.1 | PE | Invitrogen/eBioscience | 12-4714-82 |
| IgG1 Isotype | MOPC-21 | FITC | BD Pharmingen | 555748 |
| IgG2b Isotype | δG9 | PE | BD Pharmingen | 556437 |
| IgG1κ Isotype | P3.6.2.8.1 | Alexa Fluor 488 | Invitrogen/eBioscience | 53-4714-42 |
| IgG1 Isotype | MOPC-21 | PerCP Cy5.5 | BD Pharmingen | 550795 |
| IgG1 Isotype | REA293 | PE | Miltenyi Biotec | 130-118-347 |
