# Supplementary material for: The effect of COVID-19 and sex differences on natural killer cell cytotoxicity
Source: Front Cell Infect Microbiol. 2025 Sep 22;15:1635043. doi: 10.3389/fcimb.2025.1635043 (PMC12497742; doi:10.3389/fcimb.2025.1635043)
Supplement: Supplementary Table 2 — CYTOF antibody panel. [file Table2.docx]

**Supplementary Table 2. CYTOF antibody panel.**

| **Marker** | **Clone** | **Metal** | **Source** | **Catalogue #** | **Staining Dilution** |
| --- | --- | --- | --- | --- | --- |
| CD45 | HI30 | 89Y | Standard BioTools | 3089003B | 1 in 100 |
| CD19 | HIB19 | 142Nd | Standard BioTools | 3142001C | 1 in 400 |
| CD14 | HCD14 | 156Gd | Standard BioTools | 3156019C | 1 in 200 |
| CD3 | UCHT1 | 141Pr | Standard BioTools | 3141019C | 1 in 400 |
| CD8 | SK1 | 168Er | Standard BioTools | 3168002C | 1 in 400 |
| CD4 | RPA-T4 | 145Nd | Standard BioTools | 3145001C | 1 in 400 |
| CD56 | NCAM16.2 | 149Sm | Standard BioTools | 3149021C | 1 in 200 |
| CD57 | HCD57 | 176Yb | Standard BioTools | 3176019C | 1 in 100 |
| CD47 | CC2C6 | 209Bi | Standard BioTools | 3209004C | 1 in 100 |
| CD366 (TIM-3) | F38-2E2 | 159Tb | Standard BioTools | 3159037C | 1 in 100 |
| CD223 (LAG-3) | 11C3C65 | 165Ho | Standard BioTools | 3165037C | 1 in 100 |
| CD152 (CTLA4) | 14D3 | 170Er | Standard BioTools | 3170005C | 1 in 100 |
| CD159a (NKG2A) | Z199 | 169Tm | Standard BioTools | 3169013C | 1 in 100 |
| CD158b | DX27 | 173Yb | Standard BioTools | 3173010C | 1 in 100 |
| CD314 (NKG2D) | ON72 | 166Er | Standard BioTools | 3166016C | 1 in 100 |
| CD161 | HP-3G10 | 164Dy | Standard BioTools | 3164009C | 1 in 100 |
| CD279 (PD-1) | EH12.2H7 | 174Yb | Standard BioTools | 3174020C | 1 in 100 |
| Siglec 9 | K8 | 155Gd | Standard BioTools | 3155004C | 1 in 100 |
| NKB1 | DX9 | 167Er | Standard BioTools | 3167013C | 1 in 100 |
| TIGIT | MBSA43 | 154Sm | Standard BioTools | 3154016C | 1 in 100 |
| Cell-ID Ir | n/a | Ir191 & Ir193 | Standard BioTools | 201192A | 1 in 1000 |
| Cell-ID 20-Plex Pd Barcoding Kit | n/a | 102Pd, 104Pd, 105Pd, 106Pd, 108Pd, & 110Pd | Standard BioTools | 201060 | 1 in 100 |
